# Supplementary material for: Soluble urokinase plasminogen activator receptor is a prognostic biomarker in decompensated cirrhosis
Source: JHEP Rep. 2025 Nov 11;8(3):101677. doi: 10.1016/j.jhepr.2025.101677 (PMC12907094; doi:10.1016/j.jhepr.2025.101677)
Supplement: Multimedia component 1 [file mmc1.pdf]

Soluble urokinase plasminogen activator receptor is a prognostic biomarker in decompensated cirrhosis

Sven Lamatsch, Mohsin Hassan, Kai Kappert, Hilmar Berger, Qingquan Bai, Zhengyang Zhao, Nirbaanjot Walia, Carlos De La Peña-Ramirez, Raphael Mohr, Münevver Demir, Juan Wang, Fabian Artusa, Richard Sittner, Fausto Andreola, Rhea Veelken, Florian van Boemmel, Jonas Schumacher, Niklas Aehling, Janett Fischer, Rajeshwar Mookerjee, Tianhui Hu, Thomas Berg, Rajiv Jalan, Frank Tacke, Pavitra Kumar, Cornelius Engelmann

Table of contents

Supplementary Text……………………………………………………………………2

Table S1……………………………………………………………………………...9

Table S2……………………………………………………………………………10

Table S3……………………………………………………………………………11

Table S4……………………………………………………………………………15

Table S5……………………………………………………………………………16

Table S6……………………………………………………………………………17

Table S7……………………………………………………………………………18

Table S8……………………………………………………………………………19

Table S9……………………………………………………………………………22

Fig. S1……………………………………………………………………………...26

Supplementary references……………………………………………………………..27

Supplementary Text 1: Methods in detail

2.1 Study Design

In a retrospective study, circulating blood suPAR concentrations were assessed in plasma samples from patients with liver cirrhosis across two independent cohorts. The derivation cohort (n=178) consisted of healthy controls (n=6) and patients with CC (n=17), AD (n=120) liver cirrhosis, and ACLF (n=35). The validation cohort (n=197) included patients with AD (n=135) or ACLF (n=62). Plasma samples were collected upon hospital admission, with clinical data retrieved from medical records. The primary study endpoint was survival after 90 days. AD was defined as the occurrence of one or more major liver disease complications, including gastrointestinal bleeding, ascites, HE, and hepatorenal syndrome (HRS). ACLF was defined according to EASL-CLIF ACLF criteria, indicating acute decompensation of preexisting liver cirrhosis and organ failure[1]. The study adhered to good clinical practice principles and the Declaration of Helsinki (1951), with informed written consent obtained from all participants or their legal representatives before study inclusion[2].

2.2 Participating centers and patient cohort

Plasma samples and clinical data for the derivation cohort were sourced from patients hospitalized at the University College London Hospitals with acute decompensation of cirrhosis, as part of the prospective, observational DASIMAR study (Registration number: NCT01071746). This included patients with decompensated liver cirrhosis of any aetiology. The study aimed to identify biomarkers of AD and ACLF. Exclusions were made for patients with malignancies (including hepatocellular carcinoma), and individuals who had undergone major surgery, had unsolved surgical issues or were pregnant. Since the indication of

orthotopic liver transplantation (OLT) could not be determined retrospectively and the number of patients with OLT was low (n=4), all patients who underwent OLT within 90 days of the follow-up period were excluded from the analysis. For the validation cohort plasma samples and corresponding clinical data were obtained from patients with liver cirrhosis who were treated at the Leipzig University Hospital, including both outpatients and inpatients, also including patients who underwent liver transplantation and were analysed retrospectively. The presence of cirrhosis was determined by histopathological workup or non-invasive assessment of liver stiffness and typical findings on imaging, laboratory values, and medical history.

2.3 Measurement of circulating suPAR levels

Blood samples underwent centrifugation at 4,500g for 10 minutes. Heparin plasma was promptly frozen at -80° C and stored until analysis. SuPAR plasma levels were assessed using the suPARnostic® TurbiLatex (Nr. T004, suPARnostic, ViroGates, Birkerød, Denmark) on a clinical chemistry analyzer (Roche Diagnostics) at Labor Berlin – Charité Vivantes GmbH, laboratory of Charité – Universitätsmedizin Berlin, Germany. Measuring range of the test is 1.8 ng/ml to 16.0 ng/ml with a limit of detection of 1.2 ng/ml and a repeatability coefficient of variation (CV) of < 4.0% (intra-assay precision), and a CV between days of < 10% (inter-assay precision) (manufacturer performance data). Standard laboratory parameters (e.g., hemoglobin, white blood cell count (WBC), platelets, total bilirubin, creatinine, Alanine aminotransferase (ALT), Alkaline phosphatase (ALP), C-reactive protein (CRP), albumin, and International normalized ratio (INR)) were measured at the central laboratory institutions of participating centers and retrieved from patients' electronic medical records.

2.4 Mouse chronic liver disease models

All experimental protocols were conducted in compliance with local regulatory authorities' oversight and approval (approval-no: G-0174/20). Results were reported following the guidelines outlined in the ARRIVE guidelines[3]. During experiments, animals were closely monitored, and if there was a rapid deterioration in their condition, they were humanely euthanized. Euthanasia for all experimental groups was performed via exsanguination through the inferior vena cava (IVC) under general anesthesia induced by isoflurane (cp-pharma, Germany). C57B6/J mice, aged 8-10 weeks and weighing between 20 to 40 g, were obtained from the central animal facility Forschungseinrichtungen für Experimentelle Medizin (FEM) at Charité – Universitätsmedizin Berlin. Animals were randomly assigned by investigators to different intervention or control groups and were provided a standard chow diet and water. In an inflammation-triggered model, animals received intraperitoneal (i.p.) injections of 0.6 ml/kg BW CCl4 diluted in corn oil (MERCK, Germany) twice a week for 10 weeks. Three days after the final CCl4 injection, animals received an i.p. injection of 2 mg/kg BW LPS (Sigma-Aldrich, USA) derived from Klebsiella pneumonia. Animals were euthanized 24 hours after the LPS injection.

2.5 Masson's trichrome staining

Masson's trichrome staining was conducted on formalin-fixed paraffin-embedded (FFPE) 4 µm mouse liver tissue sections. The Trichrome Stain Kit (Connective Tissue Stain) from Abcam (Catalog #: ab150686) was utilized following the manufacturer's instructions. To quantify connective tissue, fluorescence microscopy imaging was performed using a ZEISS OBSERVER 7 with Colibri 7 illumination at 631/33 nm, and detection by Axiocam712 mono 12MP (4248x2832 pixel) with a pixel size of 3.1x3.1 µm, using a 20xPlnApo NA0.8 objective. Analysis of images was

conducted by measuring area fraction in FIJI 1.53o. Brightfield microscopy was conducted using a ZEISS OBSERVER 7 with Colibri 7 brightfield illumination, detection by Axiocam305 color 5MP (2464x2056 pixel) with a pixel size of 3.45x3.45 µm, using a 20xLDPln NA0.4 objective.

2.6 TUNEL staining

TUNEL staining was conducted on FFPE 4 µm mouse liver tissue sections. The in Situ Cell Death Detection Kit, POD from Roche (Catalog #: 11684817910) was utilized following the manufacturer's instructions. Brightfield microscopy was conducted using a ZEISS OBSERVER 7 with Colibri 7 brightfield illumination, detection by Axiocam305 color 5MP (2464x2056 pixel) with a pixel size of 3.45x3.45 µm, using a 20xLDPln NA0.4 objective.

2.7 Multiplex Immune Fluorescence Microscopy

Multiplex immune staining, involving repetitive immunostaining and antibody stripping, was conducted on FFPE 4 µm mouse liver tissue sections following the established facility protocol[4]. Deparaffinization and rehydration were achieved using xylene (Roth, Germany) and ethanol (Roth, Germany), respectively, with antigen retrieval performed using Tris-EDTA (pH=9). Sections underwent one-hour blocking in PBS containing 2% normal goat serum, followed by overnight incubation with primary antibodies. Staining was achieved using fluorescent dye-conjugated secondary antibodies. Details of the primary and secondary antibodies used are provided in Supplementary Table 1 and 2. Nuclear staining was performed using 4′,6-diamidino-2-phenylindole (DAPI). Antibody stripping was executed using 2-mercaptoethanol/SDS. Excitation fluorescence microscopy imaging was conducted using a ZEISS OBSERVER 7 with Colibri 7 illumination at various wavelengths, with detection by Axiocam712 mono 12MP (4248x2832 pixel) and a pixel size of 3.1x3.1

μm, using a 20xPlnApo NA0.8 objective. Image analysis, including area fraction determination, was carried out using FIJI 1.54p. For multiplex analysis, scans were aligned, hyperstacked, and concatenated using the FIJI HyperStackReg V5.6 plugin.

2.8 suPAR measurement by ELISA in murine plasma samples

Blood samples were collected from mice via exsanguination through the inferior vena cava (IVC) at the time of sacrifice and placed into tubes containing ethylenediaminetetraacetic acid (EDTA) to prevent coagulation. Following collection, the blood was centrifuged at 4,500g for 10 minutes, separating the plasma component, which was then stored at -80°C. SuPAR plasma levels were quantified using a manual enzyme-linked immunosorbent assay (ELISA) with a dilution factor of 1 to 4. The Mouse uPAR DuoSet ELISA kit from R&D Systems (Catalog #: DY531) was employed as per the manufacturer's instructions.

2.9 Single cell RNA sequencing of human liver

Liver tissue was collected during surgical intervention from two healthy donors and two patients with hepatitis B virus cirrhosis as described previously[5]. Informed consent was obtained from all the patients and donors. Liver tissue samples were quickly diluted in pre-cooled DMEM medium. Followed triple washing with PBS, tissues were cut into 1-3 mm^3 pieces, mixed with a cryopreservation solution (10% DMSO and 90% fetal bovine serum), and stored at -80°C. Aditionally single-cell transcriptomes of 5 healthy livers and 5 cirrhotic liver samples[6], and 3 cirrhotic liver samples[7] were obtained, that were described before. All datasets were processed using Seurats (v4.3.0) single-cell workflow. Cells with fewer than 200 or more than 2500 genes, or more than 15% mitochondrial content were excluded. Batch effects were minimized by using Seurat's integration anchors[8] based on 2000 highly variable

genes, and data were harmonized across batches via reciprocal principal component analysis.

2.10 Statistics

Statistical analysis was performed using SPSS® Version 29.0.0.0 (SPSS Inc., Chicago, IL). A two-sided p-value < 0.05 indicated statistical significance. For non-normally distributed values, mean and interquartile range (IQR) were calculated. Mann-Whitney U-test/Wilcoxon rank-sum test compared two independent groups, and Kruskal-Wallis test, with Bonferroni correction for multiple testing, compared multiple groups. While Spearman's rank correlation coefficient assessed the linear correlation of non-normally distributed metric or ordinal variables. Youden's J statistic was applied to the receiver operating characteristic (ROC) curve to determine the optimal threshold. Pearson chi-square test was used for testing of statistical differences between two categorical variables. To assess whether suPAR improves mortality and ACLF prediction beyond current risk stratification tools, multivariable logistic regression models were fitted with and without suPAR, and the resulting area under the curve (AUC) with their respective 95% confidence intervals (95% CI) were compared. For the derivation cohort a univariate logistic regression analysis was performed to evaluate individual variables, and those with significant associations were subsequently included in a multivariate logistic regression model. Backward elimination removed statistically insignificant variables. Additionally, a Cox proportional hazards model was used to calculate the hazard ratio over time in the validation cohort. Graphs were generated using PRISM® (GraphPad, USA) and BioRender APP (BioRender, Canada), tables were generated using Microsoft Word (Microsoft Corporation, USA).

Table S1: Primary antibodies used for Multiplex immune fluorescence staining

Target Antigen	Host	Dilution	Manufacturer	Catalog Nr.
uPAR	Rabbit	1/500	BIOSSUSA	BS-1927R
Hep Par-1	Mouse	1/500	Agilent	M715801-2
CD 45	Mouse	1/500	Agilent	M0701
CD 31	Rabbit	1/100	abcam	ab28364
IBA-1	Mouse	1/400	Sigma-Aldrich	MABN92
HNF4 alpha	Rabbit	1/1000	abcam	ab201460
CD 3	Mouse	1/200	DAKO	A0452
MPO	Rabbit	1/1000	abcam	ab208670
CK-19	Rat	1/200	DSHB biology	TROMA-III

Abbreviations: uPAR: urokinase Plasminogen Activator Receptor, Hep Par-1: Hepatocyte Specific Antigen, CD 45: Cluster of differentiation 45, Protein tyrosine phosphatase, leukocyte common antigen, CD 31: Cluster of differentiation 31, Platelet endothelial cell adhesion molecule-1, PECAM-1, IBA-1: ionized calcium-binding adapter molecule 1 (IBA1), Allograft inflammatory factor 1 (AIF-1), HNF4 alpha: Hepatocyte nuclear factor 4 alpha, CD 3: Cluster of differentiation 3, MPO: Myeloperoxidase, CK-19: Cytokeratin-19

Table S2: Secondary antibodies used for Multiplex immune fluorescence staining

Target Antigen	Fluorochrome	Host	Dilution	Manufacturer	Catalog Nr.
Mouse IgG (H+L) F(ab')2	Alexa Fluor® 488	Goat	1/500	Cell Signaling	4409
Mouse IgG (H+L) F(ab')2	Alexa Fluor® 647	Goat	1/500	Cell Signaling	4410
Rabbit IgG (H+L), F(ab')2	Alexa Fluor® 488	Goat	1/500	Cell Signaling	4412
Rabbit IgG (H+L), F(ab')2	Alexa Fluor® 647	Goat	1/500	Cell Signaling	4414
Rat IgG (H+L), F(ab')2	Alexa Fluor® 555	Goat	1/500	Cell Signaling	4417

Abbreviations: IgG: Immunoglobulin G, (H+L): heavy + light chain, F(ab')2: Antigen-binding fragment

Table S3: SuPAR levels as per organ failure in the derivation cohort

organ failure		definition	median suPAR levels	IQR suPAR levels	p-value				
					**1 - 2**	**1 - 3**	**2 - 3**		**overall**
	**liver failure**				0.002	<0.001	1.0		<0.001
	1	Bilirubin < 6 mg/dl	12.7 ng/ml	9.1 – 15.4 ng/ml					
	2	Bilirubin 6-12mg/dl	15.75 ng/ml	13.28 – 30.90 ng/ml					
	3	Bilirubin >12 mg/dl	20 ng/ml	13.90 – 31.70 ng/ml					
	**respiratory failure**								
	1	PaO2/FiO2 ratio > 300	14.00 ng/ml	10.80 – 18.33 ng/ml	0.04	0.004	0.799		0.004
	2	PaO2/FiO2 ratio 200 - 300	20.60 ng/ml	15.30 –					

				28.45 ng/ml					
	3	PaO2/FiO2 ratio ≤ 200 or mechanical ventilation	47.20 ng/ml	26.90 – 64.28 ng/ml					
	**coagulatory failure**				-	-	-		0.59
	1	INR < 2	13.60 ng/ml	10.60 – 17.20 ng/ml					
	2	INR 2 - < 2.5	20.15 ng/ml	13.45 – 24.58 ng/ml					
	3	INR ≥ 2.5	20.00 ng/ml	17.48 – 34.35 ng/ml					
	**renal failure**				0.266	0.003	0.374		0.003
	1	Creatinine < 2.0 mg/dl	14.10 ng/ml	10.80 – 18.30 ng/ml					

	2	Creatinine 2.0 - < 2.5 mg/dl	18.50 ng/ml	13.18 – 25.15 ng/ml					
	3	Creatinine ≥ 3.5 mg/dl or renal replacement therapy	38.90 ng/ml	23.40 – 64.98 ng/ml					
	**encephalopathic failure**				0.811	0.019	0.145		0.019
	1	West-Haven Grade for Hepatic Encephalopathy = 0	14.00 ng/ml	11.00 – 19.15 ng/ml					
	2	West-Haven Grade for Hepatic Encephalopathy = 1 – 2	20.30 ng/ml	14.75 – 31.35 ng/ml					
	3	West-Haven Grade for Hepatic Encephalopathy = 3 – 4	20.30 ng/ml	14.75 – 31.35 ng/ml					

	**circulatory failure**				0.014	0.008	0.639		0.008
	1	MAP ≥70 mmHg	14.55 ng/ml	11.75 – 20.60 ng/ml					
	2	MAP <70 mmHg	13.20 ng/ml	10.50 – 17.60 ng/ml					
	3	vasopressor therapy	17.90 ng/ml	11.70 – 36.40 ng/ml					

Kruskal–Wallis test with Bonferroni correction for multiple testing.

Abbreviations: suPAR: Soluble urokinase plasminogen activator receptor, IQR: Interquartile Range, PaO2, FiO2: inspiratory oxygen concentration, PaO2: arterial partial pressure of oxygen, INR: International normalized ratio, MAP: Mean Arterial Pressure

Table S4: ROC analysis for 90 days mortality in patients with decompensated cirrhosis

		AUC	CI 95%	p-value
AD score		0.800	0.660 - 0.940	0.000
MELD score		0.703	0.547 - 0.858	0.011
MELD-Na score		0.759	0.618 – 0.901	0.000
Child-Pugh Score (points)		0.721	0.599 – 0.842	0.000
SuPAR (ng/ml)		0.725	0.579 - 0.872	0.009
SuPAR ≥ 14.0 ng/ml		0.762	0.621 – 0.902	0.000
CRP (mg/dl)		0.516	0.331 – 0.702	0.864

AUROC analysis

Abbreviations: AUC: Area Under the Curve, AD score: CLIF-C Acute Decompensation score, MELD: Model for End-Stage Liver Disease, SuPAR: Soluble urokinase plasminogen activator receptor, CRP: C-reactive protein, 95% CI: 95% confidence interval, p: p-value

Table S5: ROC analysis for developing ACLF in patients with decompensated cirrhosis

		AUC	CI 95%	p-value
AD score		0.695	0.571 - 0.819	0.002
MELD score		0.740	0.617 - 0.863	0.000
MELD-Na score		0.727	0.602 – 0.852	0.000
Child-Pugh Score (points)		0.653	0.505 – 0.802	0.043
SuPAR (ng/ml)		0.673	0.528 - 0.812	0.014
SuPAR ≥ 14.7 ng/ml		0.621	0.485 – 0.758	0.081
CRP (mg/dl)		0.571	0.419 – 0.722	0.361

AUROC analysis

Abbreviations: AUC: Area Under the Curve, suPAR: Soluble urokinase plasminogen activator receptor, AD score: CLIF-C Acute Decompensation score, MELD: Model for End-Stage Liver Disease, CRP: C-reactive protein, 95% CI: 95% confidence interval, p: p-value

Table S6: Logistic regression models using a suPAR cut-off of 14.0 ng/ml for prediction of 90-day mortality in patients with decompensated liver cirrhosis in the derivation cohort

Score				Score + suPAR		
**Variables included**	**AUC**	**CI 95%**		**Variables included**	**AUC**	**CI 95%**
MELD-Na score	0.759	0.618 – 0.901		Sodium + Bilirubin + Creatinine + INR + suPAR	0.812	0.679 – 0.945
Child-Pugh Score (points)	0.728	0.572 – 0.883		Child-Pugh-Score (points) + suPAR	0.790	0.646 – 0.935
MELD	0.703	0.547 – 0.858		MELD + suPAR	0.785	0.639 – 0.931
AD Score	0.800	0.660 – 0.940		AD score + suPAR	0.843	0.728 – 0.958

AUROC analysis

Abbreviations: AUC: Area Under the Curve, MELD: Model for End-Stage Liver Disease, SuPAR: Soluble urokinase plasminogen activator receptor, 95% CI: 95% confidence interval, INR: International normalized ratio

Table S7: Logistic regression models using a suPAR cut-off of 14.7 ng/ml for prediction of ACLF in patients with decompensated liver cirrhosis in the derivation cohort

**Score**				**Score + suPAR**		
**Variables included**	**AUC**	**CI 95%**		**Variables included**	**AUC**	**CI 95%**
MELD-Na score	0.727	0.602 – 0.852		Sodium + Bilirubin + Creatinine+ INR + suPAR	0.755	0.635 – 0.876
Child-Pugh Score (points)	0.653	0.505 – 0.802		Child-Pugh-Score (points) + suPAR	0.670	0.509 – 0.830
MELD	0.740	0.617 - 0.863		MELD + suPAR	0.741	0.615 – 0.867
AD Score	0.695	0.571 - 0.819		AD score + suPAR	0.744	0.626 – 0.862

AUROC analysis

Abbreviations: AUC: Area Under the Curve, MELD: Model for End-Stage Liver Disease, SuPAR: Soluble urokinase plasminogen activator receptor, 95% CI: 95% confidence interval, INR: International normalized ratio

Table S8: Patients in the derivation cohort analysed according to patient sex

		Female (n=65)	Male (n=107)	p-value (male-female)
**Number of patients (%)**		37.8%	62.2%	
**Age (years) median (IQR)**		49 (41-59.5)	53 (46-61)	0.201
**Etiology**				0.519
	ALD (%)	32 (53.3%)	59 (57.3%)	
	viral (%)	4 (6.7%)	15 (14.6%)	
	MASLD (%)	5 (8.3%)	6 (5.8%)	
	cryptogenic (%)	1 (1.7%)	4 (3.9%)	
	multiple (%)	9 (15.0%)	13 (12.6%)	
	other/ rare (%)	9 (15.0%)	6 (5.8%)	
	no data	5 (7.7%)	4 (3.7%)	
**Laboratory and clinical parameters**				
	SuPAR (ng/ml) median (IQR)	13.6 (9.3-17.8)	14.3 (10.6-20.6)	0.314
	WBC (10^9/l) median (IQR)	8 (4.8-11.1)	8.65 (5.7-12.4)	0.365
	Platelets (10^9/l) median (IQR)	144 (85-209)	89 (60-159)	0.006
	ALT (U/l) median (IQR)	37 (25-63.5)	37 (24-73)	0.633
	ALP (U/l) median (IQR)	162 (127-220)	141 (91-210)	0.074
	Albumin (g/l) median (IQR)	30 (26-34)	30 (25-34)	0.532
	CRP (mg/dl) median (IQR)	15 (5-43)	24 (7-64)	0.162

	Hemoglobin (g/l) median (IQR)	95 (79-115)	98 (87-113)	0.334
	Sodium (mmol/l) median (IQR)	136 (132-139)	136 (131-140)	0.943
	Potassium (mmol/l) median (IQR)	3.9 (3.35-4.4)	3.8 (3.6-4.4)	0.830
	Creatinine (µmol/l) median (IQR)	69 (49.25-109.5)	76 (60-108)	0.227
	Bilirubin (µmol/l) median (IQR)	87 (25-191)	108 (39-207)	0.227
	INR median (IQR)	1.6 (1.4-1.9)	1.6 (1.4-2.0)	0.948
	MAP (mmHg) median (IQR)	79 (70-83.83)	86.67 (77-95.83)	0.019
**Disease dynamics**				
	hospitalization (days) median (IQR)	14 (8-24)	11 (6-22)	0.370
	ICU treatment	25.5%	21.6%	0.595
	90-days-mortality (%)	20%	15%	0.393

Pearson chi-square test for categorical data or the Mann–Whitney U/Wilcoxon rank-sum test for numerical data.

Abbreviations: suPAR: soluble Urokinase Plasminogen Activator Receptor, IQR: interquartile range, ALD: alcoholic liver disease, MASLD: metabolic dysfunction associated liver disease, ICU: intensive care unit, WBC: white blood cell count, ALT: alanine aminotransferase, ALP: alkaline phosphatase, CRP: C-reactive protein, ICU:

intensive care unit, INR: International normalized ratio, MAP: Mean arterial pressure, n. a.: not applicable

Table S9: Patients in the derivation cohort analyzed according to etiology of liver cirrhosis

	ALD (n=91)	viral (n=19)	MASLD (n=11)	cryptogenic (n=5)	multiple (n=22)	other / rare (n=15)	no data (n=9)	p-value (ALD-rest)
**Number of patients (%)**	55.8%	11.7%	6.7%	3.1%	13.5%	9.2%	5.2%	
**Age (years) median (IQR)**	51 (45-60)	53 (45-57)	67 (49-72)	61 (49-72)	47 (37-55)	50 (36-57)	55 (42-64)	0.985
**Laboratory and clinical parameters**								
SuPAR (ng/ml) median (IQR)	14.6 (10.9-22.7)	12.1 (6.7-20.7)	11.6 (8.1-13.7)	15.8 (8.1-16.9)	14.5 (12.2-18.1)	14.0 (12.4-18.6)	9.9 (6.0-18.5)	0.086
WBC (10^9/l) median (IQR)	8.8 (5.8-13.5)	6.9 (5.2-9.5)	7.8 (5.4-10.3)	3.8 (0.9-5.0)	7.6 (4.9-10.9)	8.9 (6.4-17.1)	9.0 (4.7-16.6)	0.100

	Platelets (10^9/l) median (IQR)	116 (75-182)	124 (65-281)	144 (61-202)	45 (13-182)	65 (42-119)	119 (100-258)	99 (60-172)	0.267
	ALT (U/l) median (IQR)	34 (23-65)	43 (31-147)	24 (12-36)	14 (11-34)	43 (27-67)	81 (49-173)	22 (14-59)	0.165
	ALP (U/l) median (IQR)	160 (115-217)	130 (80-170)	98 (75-209)	128 (47-342)	138 (108-178)	220 (130-360)	114 (67-154)	0.392
	Albumin (g/l) median (IQR)	30 (25-34)	32 (27-40)	34 (30-41)	34 (24-36)	29 (23-34)	30 (27-34)	30 (25-31)	0.420
	CRP (mg/dl) median (IQR)	25 (8-63)	26 (2-44)	17 (8-31)	43 (10-71)	9 (5-45)	15 (0-28)	13 (0-35)	0.078
	Hemoglobin (g/l) median (IQR)	95 (83-113)	107 (89-113)	115 (102-118)	79 (78-105)	98 (78-106)	110 (100-122)	95 (81-114)	0.065
	Sodium (mmol/l)	136 (131-139)	138 (132-142)	131 (128-136)	140 (136-144)	136 (130-141)	136 (130-138)	136 (130-139)	0.791

	median (IQR)								
	Potassium (mmol/l) median (IQR)	3.7 (3.4-4.4)	4.2 (3.5-4.8)	3.9 (3.5-4.7)	3.8 (3.7-4.9)	4.1 (3.6-4.4)	3.9 (3.6-4.3)	3.5 (3.2-4.1)	0.176
	Creatinine (µmol/l) median (IQR)	71 (57-100)	77 (52-159)	91 (74-105)	170 (65-102)	84 (49-121)	63 (49-92)	63 (40-168)	0.630
	Bilirubin (µmol/l) median (IQR)	102 (34-221)	128 (12-413)	33 (17-83)	39 (28-76)	84 (35-136)	168 (79-277)	73 (18-356)	0.581
	INR median (IQR)	1.7 (1.4-2.0)	1.8 (1.4-2.1)	1.5 (1.1-2.0)	1.4 (1.4-1.8)	1.8 (1.4-2.1)	1.6 (1.4-1.7)	1.6 (1.4-1.8)	0.271
	MAP (mmHg) median (IQR)	84.33 (75.66-94.66)	90 (84.66-104)	73.33 (71.33-80.66)	66.66 (62.66-78.17)	85.67 (75.17-97)	77.67 (66.67-96.67)	88.17 (83.08-99.8)	0.573
**Disease dynamics**									

	hospitalization (days) median (IQR)	10 (7-22)	14 (7-21)	15 (7-21)	16 (11-18)	17 (6-30)	14 (6-36)	12 (7-17)	0.245
	ICU treatment	25.6 %	27.3 %	20.0%	0.0%	22.2%	6.7%	42.9 %	0.220
	90-days-mortality (%)	16.5 %	10.5 %	9.1%	0.0%	22.7%	26.7 %	22.2 %	0.975

Pearson chi-square test for categorical data or the Mann–Whitney U/Wilcoxon rank-sum test for numerical data.

Abbreviations: suPAR: soluble Urokinase Plasminogen Activator Receptor, IQR: interquartile range, ALD: alcoholic liver disease, MASLD: metabolic dysfunction associated liver disease, ICU: intensive care unit, WBC: white blood cell count, ALT: alanine aminotransferase, ALP: alkaline phosphatase, CRP: C-reactive protein, ICU: intensive care unit, INR: International normalized ratio, MAP: Mean arterial pressure, n. a.: not applicable

Fig. S1: KEGG analysis for function of PLAUR in uPAR expressing cells in HBV cirrhosis

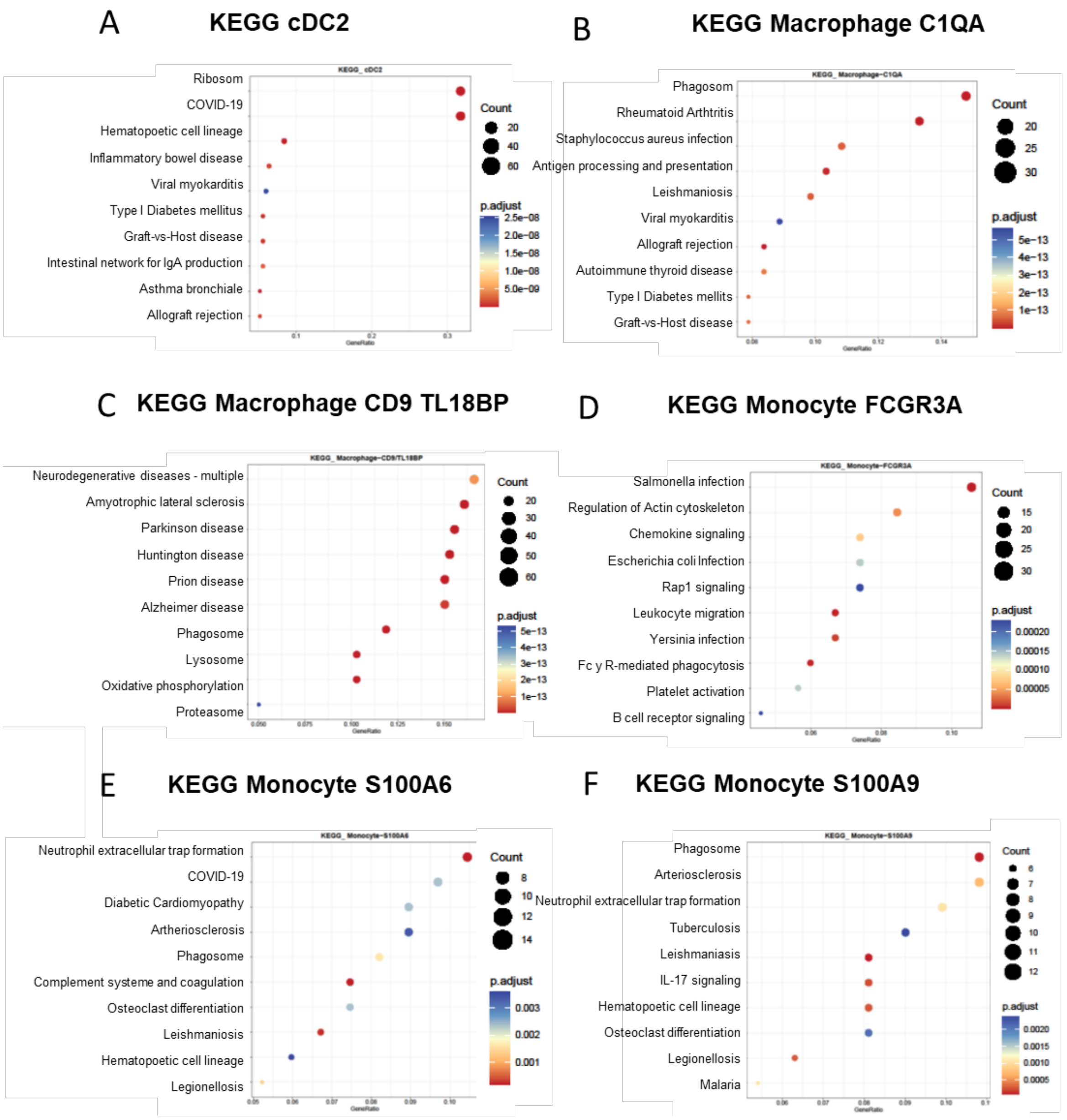

Abbreviations: KEGG: Kyoto Encyclopedia of Genes and Genomes analysis, PLAUR - Plasminogen Activator Urokinase Receptor, cDC2: Conventional Dendritic Cells, subtype 2, C1QA: complement component 1q subcomponent A, CD9: Cluster of Differentiation 9, TL18: Tetraspanin-18, TL18BP: Tetraspanin-18 binding protein, FCGR3A: Fc gamma receptor III-A (CD16a), S100A6: S100 calcium-binding protein A6, S100A9: S100 calcium-binding protein A9

Supplementary references

1. Moreau R, Jalan R, Gines P, et al. Acute-on-chronic liver failure is a distinct syndrome that develops in patients with acute decompensation of cirrhosis. *Gastroenterology*. 2013;144(7):1426-1437, 1437.e1-9. doi:10.1053/j.gastro.2013.02.042

2. WMA - The World Medical Association-WMA Declaration of Helsinki – Ethical Principles for Medical Research Involving Human Subjects. Accessed August 8, 2023. https://www.wma.net/policies-post/wma-declaration-of-helsinki-ethical-principles-for-medical-research-involving-human-subjects/

3. Percie du Sert N, Hurst V, Ahluwalia A, et al. The ARRIVE guidelines 2.0: Updated guidelines for reporting animal research. *PLoS Biol*. 2020;18(7):e3000410. doi:10.1371/journal.pbio.3000410

4. Guillot A, Kohlhepp MS, Bruneau A, Heymann F, Tacke F. Deciphering the Immune Microenvironment on A Single Archival Formalin-Fixed Paraffin-Embedded Tissue Section by An Immediately Implementable Multiplex Fluorescence Immunostaining Protocol. *Cancers*. 2020;12(9):2449. doi:10.3390/cancers12092449

5. Bai Q, Hong X, Lin H, et al. Single-cell landscape of immune cells in human livers affected by HBV-related cirrhosis. *JHEP Rep Innov Hepatol*. 2023;5(11):100883. doi:10.1016/j.jhepr.2023.100883

6. Ramachandran P, Dobie R, Wilson-Kanamori JR, et al. Resolving the fibrotic niche of human liver cirrhosis at single-cell level. *Nature*. 2019;575(7783):512-518. doi:10.1038/s41586-019-1631-3

7. Zhang Q, He Y, Luo N, et al. Landscape and Dynamics of Single Immune Cells in Hepatocellular Carcinoma. *Cell*. 2019;179(4):829-845.e20. doi:10.1016/j.cell.2019.10.003

8. Stuart T, Butler A, Hoffman P, et al. Comprehensive Integration of Single-Cell Data. *Cell*. 2019;177(7):1888-1902.e21. doi:10.1016/j.cell.2019.05.031
