# Supplementary material for: Soluble urokinase plasminogen activator receptor is a prognostic biomarker in decompensated cirrhosis
Source: JHEP Rep. 2025 Nov 11;8(3):101677. doi: 10.1016/j.jhepr.2025.101677 (PMC12907094; doi:10.1016/j.jhepr.2025.101677)
Supplement: Multimedia component 2 [file mmc2.docx]

**JHEP Reports**

**CTAT methods**

Tables for a “Complete, Transparent, Accurate and Timely account” (CTAT) are now mandatory for all revised submissions. The aim is to enhance the reproducibility of methods.

- Only include the parts relevant to your study
- Refer to the CTAT in the main text as ‘Supplementary CTAT Table’
- Do not add subheadings
- Add as many rows as needed to include all information
- Only include one item per row

**If the CTAT form is not relevant to your study, please outline the reasons why:**

|  |
| --- |

- 1. **Antibodies**

| **Name** | **Dilution** | **Supplier** | **Cat no.** | **Clone no.** |
| --- | --- | --- | --- | --- |
| uPAR | 1:500 | BIOSSUSA | BS-1927R | **-** |
| Hep Par-1 | 1:500 | Agilent | M715801-2 | OCH1E5 |
| CD 45 | 1:500 | Agilent | M0701 | 2B11 + PD7/26 |
| CD 31 | 1:100 | abcam | ab28364 | **-** |
| IBA-1 | 1:400 | Sigma-Aldrich | MABN92 | 20A12.1 |
| HNF4 alpha | 1:1000 | abcam | ab201460 | EPR16885-99 |
| CD 3 | 1:200 | DAKO | A0452 | **-** |
| MPO | 1:1000 | abcam | ab208670 | EPR20257 |
| CK-19 | 1:200 | DSHB biology | MABT913 | TROMA-3 |
| Mouse Alexa Fluor® 488 | 1:500 | Cell Signaling | 4409 | **-** |
| Mouse Alexa Fluor® 647 | 1:500 | Cell Signaling | 4410 | **-** |
| Rabbit Alexa Fluor® 488 | 1:500 | Cell Signaling | 4412 | **-** |
| Rabbit Alexa Fluor® 647 | 1:500 | Cell Signaling | 4414 | **-** |
| Rat Alexa Fluor® 555 | 1:500 | Cell Signaling | 4417 | **-** |

- 1. **Cell lines**

| **Name** | **Citation** | **Supplier** | **Cat no.** | **Passage no.** | **Authentication test method** |
| --- | --- | --- | --- | --- | --- |
| NA | NA | NA | NA | NA | NA |

- 1. **Organisms**

| **Name** | **Supplier** | **Strain** | **Sex** | **Age** | **Overall n number** |
| --- | --- | --- | --- | --- | --- |
| Wildtype Mice | Forschungseinrichtungen für Experimentelle Medizin (FEM) Charité – Universitätsmedizin Berlin | C57B6/J | m | 18-25 weeks | 15 |

- 1. **Sequence based reagents**

| **Name** | **Sequence** | **Supplier** |
| --- | --- | --- |
| NA | NA | NA |

- 1. **Biological samples**

| **Description** | **Source** | **Identifier** |
| --- | --- | --- |
| Human Plasma | Liver patients and healthy subjects | Plasma |

- 1. **Deposited data**

| **Name of repository** | **Identifier** | **Link** |
| --- | --- | --- |
| NA | NA | NA |

- 1. **Software**

| **Software name** | **Manufacturer** | **Version** |
| --- | --- | --- |
| SPSS® | SPSS Inc., Chicago, IL | 29.0.0.0 |
| Microsoft Word 2019 | Microsoft Corporation, USA | 1808 |
| PRISM® | GraphPad, USA | 8.4.3 |
| BioRender APP | BioRender, Canada | 02.10.2024 |
| FIJI | National Institutes of Health, USA | 1.54p |
| ZEN blue | Carl Zeiss Microscopy GmBH, Germany | 3.1 |

- 1. **Other (*e.g*. drugs, proteins, vectors etc.)**

| NA | NA | NA |
| --- | --- | --- |
|  |  |  |

- 1. **Please provide the details of the corresponding methods author for the manuscript:**

| Prof. Dr. med. Cornelius Engelmann MD/PhD  Email: [cornelius.engelmann@charite.de](mailto:cornelius.engelmann@charite.de); Tel.: +49-30-450-553022; Fax: +49-30-450-553902; Charité Universitätsmedizin Berlin Campus-Virchow-Klinikum Augustenburger Platz 1, 13353 Berlin; Intern: Forum 4, Raum 2.0704a |
| --- |

**2.0 Please confirm for randomised controlled trials all versions of the clinical protocol are included in the submission. These will be published online as supplementary information.**

| NA |
| --- |
