# Supplementary material for: Soluble urokinase plasminogen activator receptor is a prognostic biomarker in decompensated cirrhosis
Source: JHEP Rep. 2025 Nov 11;8(3):101677. doi: 10.1016/j.jhepr.2025.101677 (PMC12907094; doi:10.1016/j.jhepr.2025.101677)
Supplement: Multimedia component 4 [file mmc4.pdf]

Soluble urokinase plasminogen activator receptor is a prognostic biomarker in decompensated cirrhosis

Sven Lamatsch[1], Mohsin Hassan[1], Kai Kappert[2,3], Hilmar Berger[1], Qingquan Bai[1,4], Zhengyang Zhao[4], Nirbaanjot Walia[1], Carlos De La Peña-Ramirez[5], Raphael Mohr[1], Münevver Demir[1], Juan Wang[1], Fabian Artusa[1], Richard Sittner[1], Fausto Andreola[6], Rhea Veelken[7], Florian van Boemmel[7], Jonas Schumacher[7], Niklas Aehling[7,8], Janett Fischer[7], Rajeshwar Mookerjee[6], Tianhui Hu[4], Thomas Berg[7], Rajiv Jalan[6], Frank Tacke[1], Pavitra Kumar[1,‡], Cornelius Engelmann[1,6,*,‡]

JHEP Reports **2026**. vol. 8 | 1–13

Check for updates

Background & Aims: Cirrhosis poses a significant healthcare burden, with decompensation and acute-on-chronic liver failure (ACLF) resulting in high morbidity and mortality. Reliable biomarkers of disease progression are urgently needed. Urokinase plasminogen activator receptor (uPAR) and its soluble form (suPAR) are linked to systemic inflammation in liver disease. This study aims to evaluate suPAR as a prognostic marker and its role in chronic liver disease.

Methods: SuPAR levels were measured in a derivation cohort (n = 178) and a validation cohort (n = 197) from two centers, including healthy controls and patients with cirrhosis, acute decompensation, and ACLF. In a mouse model using carbon tetrachloride and lipopolysaccharide, suPAR levels correlated with liver uPAR expression. Single-cell RNA sequencing was used to analyze uPAR expression in immune cells from healthy controls and from patients with HBV-related cirrhosis.

Results: SuPAR levels correlated with disease severity markers, including creatinine, bilirubin, albumin, international normalized ratio, MELD score, and hospitalization duration (all p <0.001). They were associated with higher in-hospital mortality (p = 0.02), intensive care unit treatment (p <0.001), 90-day mortality (p = 0.003), and ACLF progression (p = 0.014). SuPAR levels ≥14.0 ng/ml independently predicted 90-day mortality in decompensated cirrhosis (hazard ratio [HR] 5.295, p = 0.015). The validation cohort confirmed these correlations, with increased 28-day (HR 9.589, p <0.001) and 90-day (HR 7.899, p <0.001) mortality. In mice, suPAR and liver uPAR expression were significantly higher in acute-on-chronic injury compared with chronic injury and control groups. Single-cell RNA sequencing in human liver immune cells revealed increased PLAUR expression in monocytes, macrophages, and dendritic cells in HBV-induced cirrhosis.

Conclusions: SuPAR is a potential biomarker for predicting outcomes in acute decompensation, reflecting both systemic and liver-specific inflammation. Further studies are needed to clarify the role of uPAR-expressing cells in disease progression.

© 2025 The Authors. Published by Elsevier B.V. on behalf of European Association for the Study of the Liver (EASL). This is an open access article under the CC BY license (http://creativecommons.org/licenses/by/4.0/).

Introduction

Chronic liver diseases (CLD) present multifaceted global health challenges, characterized by progressive hepatic dysfunction and associated complications such as acute decompensated cirrhosis (AD) and acute-on-chronic liver failure (ACLF).[2] AD commonly manifests with clinical features including ascites, hepatic encephalopathy, and variceal bleeding, predominantly precipitated by bacterial infections or alcohol-associated hepatitis.[3] ACLF represents a critical syndrome marked by the abrupt decompensation of pre-existing CLD, systemic inflammation, deteriorated hepatic function, and multi-organ failure. These specific complications are associated with poor prognosis and limited therapeutic options, with liver transplantation remaining the only definitive intervention.[3] Despite advances in understanding CLD pathophysiology, the search for reliable prognostic markers is of critical importance.

Prognostic biomarkers may specifically predict the future clinical trajectory, and many have previously been evaluated for their ability to predict outcomes in liver diseases.[4] Existing scoring systems, *e.g.* model for end-stage liver disease (MELD), provide valuable insights but may not fully capture the complexities of the disease. Therefore, the pursuit of novel, non-invasive biomarkers is imperative, especially given the lack of reliability in patients with ACLF.

The urokinase plasminogen activator receptor (uPAR) is a cell surface receptor protein that binds to the urokinase plasminogen activator, a serine protease involved in regulating fibrinolysis and cell migration. Preclinical investigations in animal models have delineated the pivotal role of uPAR in hepatic fibrogenesis, with uPAR-deficient models exhibiting resistance to fibrotic progression and tissue damage.[5,6] Its soluble form, soluble urokinase plasminogen activator receptor (suPAR), is found in the blood

* Corresponding author. Address: Charité Universitätsmedizin Berlin Campus-Virchow-Klinikum Augustenburger Platz 1, 13353 Berlin; Intern: Forum 4, Raum 2.0704a, Germany; Tel.: +49-30-450-553022; Fax: +49-30-450-553902.
E-mail address: cornelius.engelmann@charite.de (C. Engelmann).
‡ Authors share the last authorship
https://doi.org/10.1016/j.jhepr.2025.101677

and shed from various cells, including immune, endothelial, and certain cancer cells. In the milieu of CLD, increased levels of proinflammatory cytokines such as Interleukin (IL)-6, IL-8, and IL-1 receptor antagonists, juxtaposed with anti-inflammatory mediators like IL-10, highlight the coexistence of systemic inflammation and immunoparalysis.[7] This gradient of inflammation is a pivotal determinant of clinical prognosis, with subclinical systemic inflammation precipitating complications and AD, while pronounced systemic inflammation acts as the main mediator of organ failure.[8] Elevated suPAR levels have been associated with various inflammatory conditions, prompting investigations into its potential utility in CLD.[9–11] It has been shown that is elevated in patients with CLD and predicts prognosis.[11,12] In patients with hepatitis B-related ACLF, suPAR levels correlated with 30- and 90-day mortality independently of the SOFA (sequential organ failure assessment) and MELD scores.[13] However, a validated analysis of the prognostic value for progression from AD of CLD to ACLF and pathophysiological understanding are still missing. In summary, robust prognostic biomarkers are urgently needed in end-stage CLD to optimize therapeutic strategies, and suPAR shows promise as one such biomarker.

In this study, we examined two diverse cohorts, including patients with compensated and decompensated cirrhosis, as well as ACLF, and integrated clinical data with insights from murine models of ACLF to investigate suPAR as a prognostic marker in CLD.

Methods

Study design

We conducted a retrospective study measuring plasma suPAR levels in two independent cohorts of patients with cirrhosis. The derivation cohort (n = 178) included healthy controls (n = 6) and patients with compensated cirrhosis (CC, n = 17), AD (n = 120), and ACLF (n = 35). The validation cohort (n = 197) comprised patients with AD (n = 135) and ACLF (n = 62). Plasma was collected at hospital admission, and the primary endpoint was 90-day survival. AD was defined by major complications (ascites, gastrointestinal bleeding, HE, HRS), and ACLF by EASL-CLIF criteria.[14] All patients or legal representatives provided informed consent.[15]

Patient cohorts

Derivation cohort data were obtained from the prospective DASIMAR study (NCT01071746) at University College London Hospitals, including patients with decompensated cirrhosis of any etiology. Exclusion criteria were malignancy, major surgery, pregnancy, or liver transplantation within 90 days. The validation cohort included out- and in-patients from Leipzig University Hospital. Cirrhosis was confirmed by histology, liver stiffness, or typical imaging/Laboratory findings.

suPAR measurement

Blood samples were centrifuged (4,500 g, 10 min), plasma stored at −80 °C, and suPAR measured using the suPARnostic® TurbiLatex assay (ViroGates, Denmark) with a detection limit of 1.2 ng/ml and inter-assay CV <10%. Standard labs (*e.g.* bilirubin, creatinine, international normalized ratio, C-reactive protein [CRP]) were obtained from routine hospital testing.

Mouse models

C57BL/6J mice (8–10 weeks) were treated with carbon tetrachloride (CCl_4) for 10 weeks to induce chronic injury and then challenged with lipopolysaccharide (LPS) to model inflammation-triggered decompensation. Mice were euthanized 24 h after LPS. All procedures complied with local ethical regulations (approval-no: G-0174/20) and were reported following the ARRIVE guidelines.[16]

Histology & imaging

Liver tissue sections were processed for Masson's trichrome staining (Catalog number: ab150686), TUNEL staining (Catalog number: 11684817910), and multiplex immunofluorescence[17] using standard protocols. Imaging was performed with ZEISS OBSERVER 7 microscopes, and analyses (*e.g.* area fraction, image alignment) were conducted with FIJI software.

suPAR in murine plasma

Mouse plasma was collected at sacrifice and suPAR measured by ELISA (R&D Systems, DY531).

Single-cell RNA sequencing

Liver tissue was collected from healthy donors and patients with cirrhosis.[18–20] Additional published single-cell RNA sequencing datasets were integrated using Seurat (v4.3.0). Cells with <200 or >2,500 genes or >15% mitochondrial content were excluded, and batch correction[21] was applied via reciprocal principal component analysis.

Statistics

Analyses were performed in SPSS v29. Non-parametric tests (Mann–Whitney, Kruskal–Wallis with Bonferroni correction) and Spearman's correlation were applied. ROC analyses used Youden's J statistic. Multivariable logistic regression models assessed suPAR's prognostic value for mortality/ACLF compared with existing tools. Cox regression was used for survival analysis in the validation cohort. Graphs were generated with GraphPad PRISM and BioRender.

For detailed materials & methods, see the supplementary information and supplementary CTAT table.

Results

Baseline patient characteristics – derivation cohort

The cohort was predominantly male (60.1%), with a median age of 51 years (IQR 43.25–61). Alcohol-related liver disease was the primary etiology, followed by viral hepatitis. The 90-day mortality rates were 13.3% for patients with AD and 37.1% for patients with ACLF. Six healthy individuals were included as healthy controls. For baseline patient characteristics see Table 1.

suPAR levels correlate with disease severity

We first investigated differences in suPAR levels across stages of cirrhosis. In our derivation cohort, median suPAR was 1.95 ng/ml (IQR 1.6–2.5 ng/ml) in healthy controls and 6.5 ng/ml (IQR 4.7–10.8 ng/ml) in patients with CC, without significant difference (p = 0.835). Patients with AD had

Table 1. Patient characteristics at baseline.

	Derivation cohort					Validation cohort		
	Compensated cirrhosis	Decompensated cirrhosis	ACLF	*p* value	*p* value	Decompensated cirrhosis	ACLF	*p* value
	(n = 17)	(n = 120)	(n = 35)	CC - AD	AD - ACLF	(n = 135)	(n = 62)	AD - ACLF
Sex				0.135	0.138			<0.001
Female (%)	4 (23.5%)	51 (42.5%)	10 (28.6%)			46 (34.1%)	11 (17.8%)	
Male (%)	13 (76.5%)	69 (57.5%)	25 (71.4%)			89 (65.9%)	51 (82.2%)	
Age (years) median (IQR)	56 (51-67)	51 (42-61)	50 (43-57)	0.081	1.0	57 (51-65)	56 (51-64.25)	0.584
Etiology				0.001	0.201			<0.001
ALD (%)	4 (23.5%)	67 (55.8%)	20 (57.1%)	0.06	n. a.	70 (51.9%)	45 (72.6%)	0.02
Viral (%)	6 (35.3%)	9 (7.5%)	4 (11.5%)	0.001	n. a.	20 (14.8%)	2 (3.2%)	n. a.
MASLD (%)	5 (29.4%)	6 (5.0%)	0 (0.0%)	0.001	n. a.	19 (14.1%)	5 (8.1%)	0.04
Cryptogenic (%)	0 (0.0%)	2 (1.7%)	3 (8.6%)	0.58	n. a.	6 (4.4%)	6 (9.7%)	1.0
Multiple (%)	2 (11.8%)	16 (13.3%)	4 (11.4%)	0.790	n. a.	6 (4.4%)	2 (3.2%)	n. a.
Other/rare (%)	0 (0.0%)	13 (10.8%)	2 (5.7%)	0.140	n. a.	14 (10.4%)	2 (3.2%)	n. a.
No data	0 (0.0%)	7 (5.8%)	2 (5.7%)	0.307	n. a.	0 (0.0%)	0 (0.0%)	n. a.
Laboratory and clinical parameters								
SuPAR (ng/ml) median (IQR)	6.5 (4.7-10.8)	13.7 (10.65-17.825)	20.0 (14.1-36.0)	<0.001	<0.001	11.0 (7.2-15.5)	21.1 (14.975-27.025)	<0.001
WBC (10-9/L) median (IQR)	6.7 (3.7-7.4)	8.0 (5.2-11.5)	10.0 (5.6-16.3)	0.006	0.092	6.1 (4.9-8.0)	9.5 (6.9-14.4)	<0.001
Platelets (10-9/L) median (IQR)	122 (88-215)	114 (71-176)	93 (60-194)	0.324	0.329	136 (90-187)	96 (57-152)	0.007
ALT (U/L) median (IQR)	34 (21-53)	37 (23-53)	60 (28-120)	0.170	0.107	34 (23-62)	29 (22-53)	0.335
ALP (U/L) median (IQR)	95 (60-134)	149 (112 -213)	156 (99-225)	0.009	0.981	120 (79-165)	152 (109-240)	0.007
Albumin (g/L) median (IQR)	43 (35-44)	30 (26-34)	29 (25-34)	<0.001	0.455	39 (34-45)	30 (26-35)	<0.001
CRP (mg/dl) median (IQR)	2 (0-5)	15 (5-44)	36 (11-71)	<0.001	0.011	6 (2-19)	40 (21-60)	<0.001
Hemoglobin (g/L) median (IQR)	134 (123-148)	100 (85-114)	91 (81-106)	<0.001	0.145	124 (106-147)	92 (77-101)	<0.001
Sodium (mmol/L) median (IQR)	140 (137-142)	136 (132-139)	135 (128-141)	0.021	0.584	138 (135-140)	135 (132-140)	0.029
Potassium (mmol/L) median (IQR)	4.2 (4.2-4.3)	3.8 (3.6-4.4)	4.0 (3.55-5.0)	0.391	0.170	4.3 (3.9-4.6)	4.3 (3.8-4.8)	0.677
Creatinine (µmol/L) median (IQR)	80 (68-85)	65 (51-92)	191 (93-292)	<0.001	<0.001	73 (61-90)	187 (116-281)	<0.001
Bilirubin (µmol/L) median (IQR)	12 (7-27)	84 (30-158)	176 (70-427)	<0.001	0.004	20 (13-35)	73 (35-193)	<0.001
INR median (IQR)	-	1.6 (1.4-1.9)	1.8 (1.5-2.3)	-	0.010	1.2 (1.1-1.4)	1.7 (1.3-2.3)	<0.001
MAP (mmHg) median (IQR)	100 (83-108)	85 (75-97)	82 (70-89)	0.003	0.195	85 (77-96)	79 (72-86)	0.010
Disease dynamics								
Hospitalization (days) median (IQR)	-	10.0 (6.0-19.75)	17.0 (13.0-28.0)		0.001	0 (0-1.5)	3 (1.0-14.25)	<0.001
ICU treatment	-	15.6%	45.7%		<0.001	0%	32.1%	<0.001
Mortality								
90-day mortality (%)	0%	13.3%	37.1%	0.178	0.01	4.4%	41.9%	<0.001
Scores								
CLIF-C AD score median (IQR)	n. a.	52.1 (45.5-57.3)	n. a.		-	47 (42-52)	n. a.	-
CLIF-C ACLF score median (IQR)	n. a.	n. a.	46.7 (40.3-54.3)		-	n. a.	44 (40.5-53.5)	-
MELD median (IQR)	-	18.5 (14.0-21.0)	29.0 (22.75-34.5)		<0.001	12.0 (9.0-17.0)	25.5 (21.0-30.25)	<0.001
CPS (points) median (IQR)	-	9.0 (8.0-11.0)	11.0 (10.0-12.0)		<0.001	7.0 (6.00-7.0)	9.0 (8.0-10.25)	<0.001
ACLF grade								
ACLF grade 1	-	-	38.2 %			-	71%	-
ACLF grade 2	-	-	50%			-	21%	-
ACLF grade 3	-	-	11.8%			-	8%	-
Organ failure								

(*continued on next page*)

Table 1. (*continued*)

	Derivation cohort					Validation cohort		
	Compensated cirrhosis	Decompensated cirrhosis	ACLF	*p* value	*p* value	Decompensated cirrhosis	ACLF	*p* value
	(n = 17)	(n = 120)	(n = 35)	CC - AD	AD - ACLF	(n = 135)	(n = 62)	AD - ACLF
Liver failure	-	18.0%	44.1%					
Respiratory failure	-	0.8%	8.6%					
Coagulation failure	-	4.5%	20.6%					
Renal failure	-	0.0%	58.8%					
Brain failure	-	2.5%	20.6%					
Circulatory failure	-	5.0%	20.0%					

Baseline characteristics of the derivation and validation cohorts by admission status, are presented as mean and interquartile range (IQR). Statistical significance was assessed using Pearson chi-square test for categorical data or Mann–Whitney *U*/Wilcoxon rank-sum test for numerical data. AD, acute decompensation; ACLF, acute-on-chronic liver failure; ALD, alcoholic liver disease; ALP, alkaline phosphatase; ALT, alanine aminotransferase; CC, compensated cirrhosis; CPS, Child-Pugh score; ICU, intensive care unit; INR, international normalized ratio; MAP, mean arterial pressure; MASLD, metabolic dysfunction–associated liver disease; MELD, model for end-stage liver disease; n.a., not applicable; suPAR, soluble urokinase plasminogen activator receptor.

significantly higher suPAR levels (13.7 ng/ml, IQR 10.7–17.8 ng/ml) than both controls (p <0.001) and patients with CC (p = 0.001). However, AD suPAR levels were significantly lower (p = 0.001) than those observed in patients with ACLF (20.0 ng/ml, IQR 14.1–36.8 ng/ml). Within ACLF, grade 1 patients had lower suPAR than grade 2 (p = 0.006) and grade 3 (p = 0.034) patients. Levels in grade 2 were significantly higher than in AD (p <0.001), while no difference was observed between AD and ACLF grade 1 (p = 1) or between ACLF grades 2 and 3 (p = 1) (Fig. 1A,B).

Next, individual organ failures, as defined by the CLIF-C organ failure (CLIF-C OF) score, were analyzed. Grade 3 failure of the liver, kidney, lungs, coagulation system, and brain was associated with significantly higher serum suPAR levels compared with grade 1 (liver: p <0.001; kidney: p = 0.003; lungs: p = 0.008; coagulation: p = 0.004; brain: p = 0.019), whereas circulatory failure showed no significant association (p = 0.590) (Fig. 1C–H). Serum suPAR levels increased with early dysfunction in the liver, lungs, and coagulation system, but only rose in later stages of renal and brain failure, and remained unchanged in circulatory failure. This suggests a potential organ-specific link to cytotoxicity, though the underlying mechanisms remain unclear.

SuPAR also correlated with established biochemical markers of disease severity (Fig. 2A,B). Significant associations were found with creatinine (r = 0.209, p = 0.012), bilirubin (r = 0.473, p <0.001), international normalized ratio (r = 0.323, p <0.001), alanine aminotransferase (r = 0.273, p = 0.001), and albumin (negative correlation, r = –0.247, p = 0.02). A weaker correlation was observed with CRP (r = 0.184, p = 0.028), but not with white blood cell count (r = 0.147, p = 0.074).

Further, suPAR showed strong positive correlations with composite disease severity scores. Associations were significant with the MELD score (r = 0.486, p <0.001) and the CLIF-C ACLF score (r = 0.611, p <0.001), which integrates organ failure, inflammation, and age. No correlation was seen with the CLIF-C AD score (r = 0.102, p = 0.298) (Fig. 2C–E), likely reflecting the lack of association between suPAR and white blood cell count. These findings emphasize suPAR as a marker of disease progression and prognosis in CLD.

Finally, we examined whether elevated suPAR reflected infection. In the derivation cohort, 18.8% of patients presented with acute infection, with comparable rates in AD (18.4%) and ACLF (20.0%) (p = 0.809). Baseline suPAR did not differ significantly between those with infection (14.2 ng/ml, IQR 10.95–19.9) and those without (16.1 ng/ml, IQR 13.6–24.6) (p = 0.117). However, suPAR significantly predicted incident infections during hospitalization: patients who developed infections had higher baseline suPAR (18.3 ng/ml, IQR 14.8–29.7) than those who remained infection-free (13.3 ng/ml, IQR 10.5–15.4; p <0.001).

SuPAR is associated with adverse outcomes and independently indicates prognosis in decompensated cirrhosis

We next assessed whether suPAR levels predicted clinical outcomes. Among patients with AD at admission, survivors or those lost to follow-up had lower plasma suPAR (median 13.40 ng/ml, IQR 10.5–16.8) compared to those who died within 90 days (15.15 ng/ml, IQR 14.2–20.8; p = 0.039)

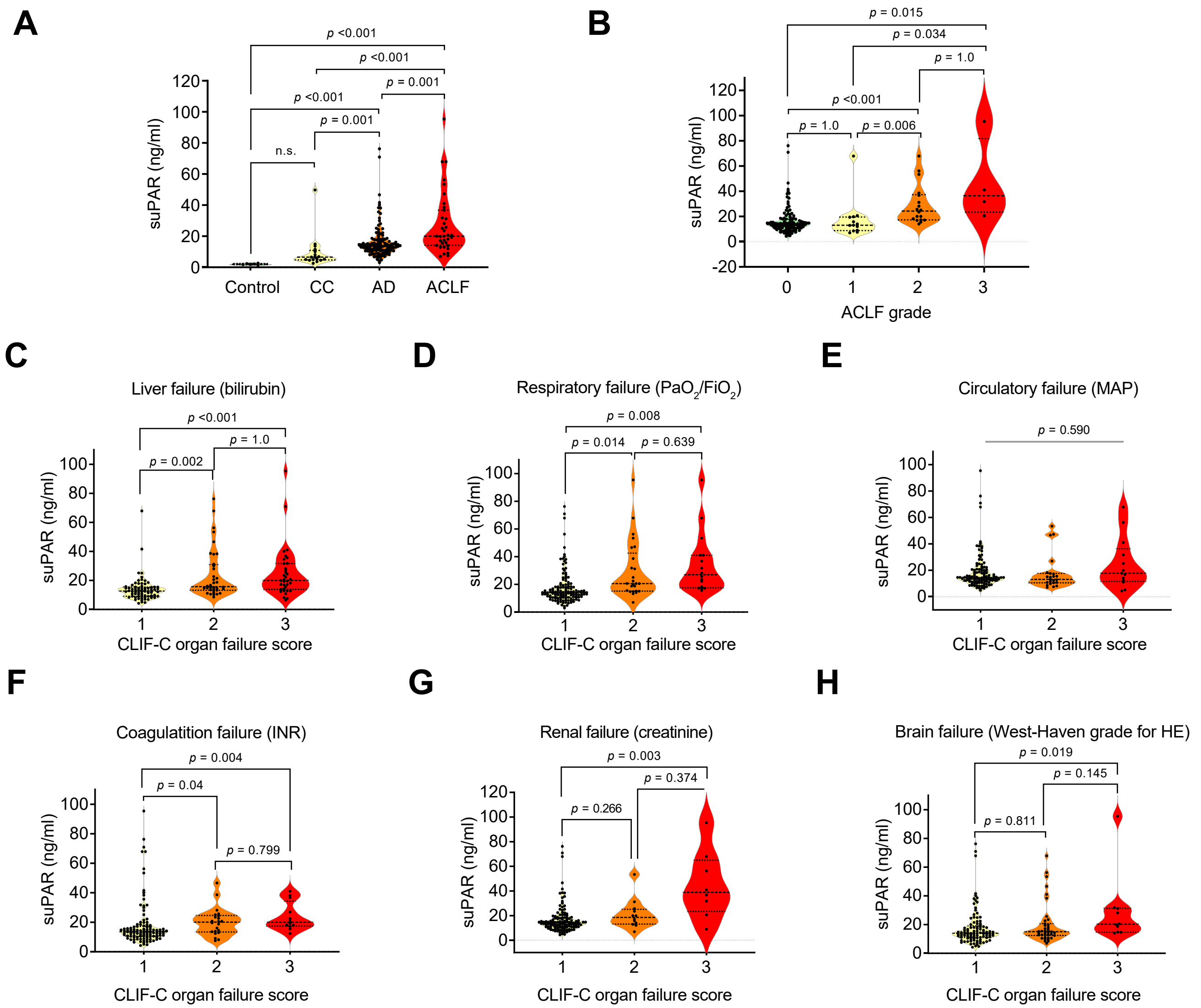

Fig. 1. SuPAR plasma levels as per ACLF grade and CLIF-C-OF score in the derivation cohort. Kruskal–Wallis Test with Bonferroni correction. (A) Plasma suPAR levels at admission differed by clinical status. Median levels were 1.95 ng/ml (IQR 1.63–2.45) in healthy controls, 6.5 ng/ml (IQR 4.7–10.8) in compensated cirrhosis (CC), 13.7 ng/ml (IQR 10.65–17.83) in acute decompensation (AD), and 20.0 ng/ml (IQR 14.1–36.8) in acute-on-chronic liver failure (ACLF) (p = 0.001 *vs.* CC; p <0.001 *vs.* controls). (B) By ACLF grade, suPAR did not differ between AD and ACLF grade 1 (p = 1.0) or between grades 2 and 3 (p = 1.0). Significant differences were observed between ACLF grade 0 *vs.* 2 (p <0.001), grade 1 *vs.* 2 (p = 0.006), grade 0 *vs.* 3 (p = 0.015), and grade 1 *vs.* 3 (p = 0.034). Organ-specific analyses showed variable patterns: (C) Liver failure – suPAR increased from grade 1 to 2 (p = 0.002) with no further rise in grade 3 (p = 1.0). (D) Respiratory failure – increased from grade 1 to 2 (p = 0.014), with no further rise in grade 3 (p = 0.639). (E) Circulatory failure – no significant differences between grades 1–3 (p = 0.590). (F) Coagulation failure – increased from grade 1 to 2 (p = 0.040), with no additional increase in grade 3 (p = 0.799). (G) Renal failure – elevation observed only in advanced stages; grade 1 *vs.* 3 (p = 0.003). No differences between grade 1 *vs.* 2 (p = 0.266) or 2 *vs.* 3 (p = 0.374). (H) Brain failure – suPAR elevated in severe stages; grade 1 *vs.* 3 (p = 0.019). No significant changes between grade 1 *vs.* 2 (p = 0.811) or 2 *vs.* 3 (p = 0.145). ACLF, acute-on-chronic liver failure; AD, acute decompensation; CC, compensated cirrhosis; INR, international normalized ratio; MAP, mean arterial pressure; PaO_2/FiO_2, partial pressure of oxygen/inspiratory oxygen fraction; suPAR, soluble urokinase plasminogen activator receptor.

(Fig. 3A). In ACLF, median suPAR did not differ between survivors or those lost to follow-up (20.0 ng/ml, IQR 12.98–32.6) and deceased patients (20.7 ng/ml, IQR 17.7–39.8; p = 0.468) (Fig. 3B), nor did in-hospital mortality (p = 0.440). However, patients with ACLF admitted to the intensive care unit (ICU) had higher suPAR (22.9 ng/ml, IQR 19.5–40.4) than those not admitted (16.6 ng/ml, IQR 12.7–26.3; p = 0.050). SuPAR also correlated positively with hospitalization duration (r = 0.361, p <0.001).

In ROC analysis for 90-day mortality in AD, suPAR achieved an AUC of 0.725 (95% CI 0.579–0.872) with an optimal cut-off of 14.0 ng/ml (sensitivity 0.813, specificity 0.711) (Fig. 3D). This performance was compared with MELD (AUC 0.703, 95% CI 0.547–0.858), CLIF-C AD score (AUC: 0.800, 95% CI 0.660–0.940), and CRP (AUC: 0.516, 95% CI 0.331–0.702). Patients with suPAR ≥14.0 ng/ml had higher in-hospital mortality (20.7% *vs.* 7.6%, p = 0.024), more ICU admissions (20.7% *vs.* 8.1%, p = 0.049), and increased 90-day mortality (22.4% *vs.* 4.8%, p = 0.005). In multivariate analysis, suPAR ≥14.0 ng/ml (odds ratio 5.295, 95% CI 1.377–20.362, p = 0.015) and sodium (odds ratio 0.912, 95% CI 0.832–0.999, p = 0.049) independently predicted 90-day mortality (Table 2). To determine

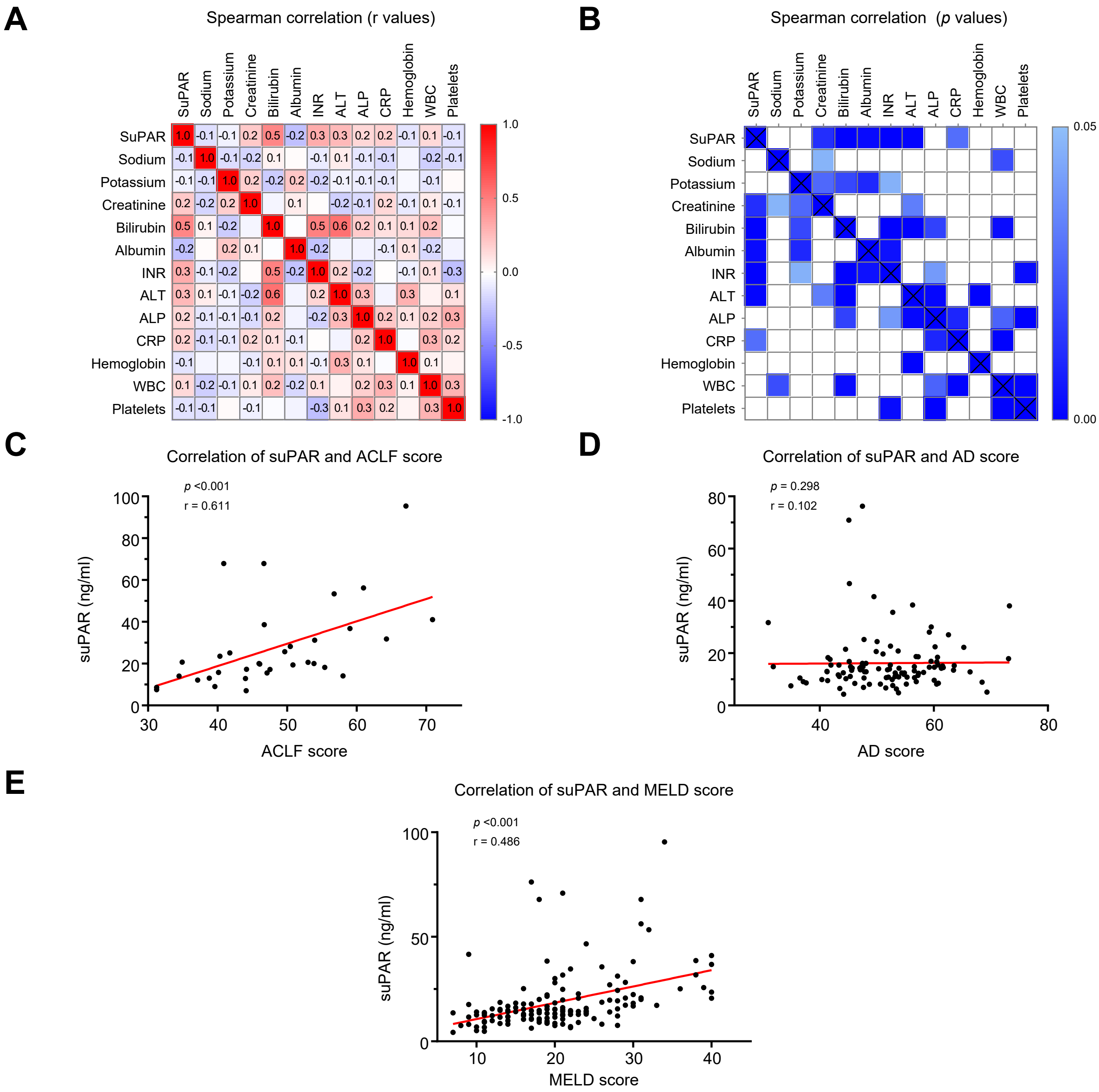

Fig. 2. Correlation of suPAR with clinical parameters and outcome scores. Correlation (Spearman correlation coefficient) of suPAR with clinical parameters and outcome scores in the derivation cohort. (A,B) Correlation (Spearman) between suPAR and biochemical markers: r-values (A) are depicted on the left, with red indicating a positive correlation and blue indicating a negative correlation and *p* values (B) are shown on the right, with statistically significant correlations color-coded in blue. SuPAR levels were significantly correlated with creatinine (r = 0.209, *p* <0.012), bilirubin (r = 0.473, *p* <0.001), albumin (r = -0.247, *p* <0.02), INR (r = 0.323, *p* <0.001), ALT (r = 0.273, *p* = 0.001), and CRP (r = 0.184, *p* = 0.028). (C) SuPAR levels were significantly correlated with the CLIF-C ACLF score in patients with ACLF (r = 0.611, *p* <0.001). (D) SuPAR levels were not significantly correlated with the CLIF-C AD score in patients with AD (r = 0.102, *p* = 0.298). (E) SuPAR levels were significantly correlated with the MELD score (r = 0.486, *p* <0.001). ACLF, acute-on-chronic liver failure; AD, acute decompensation; ALP, alkaline phosphatase; ALT, alanine aminotransferase; CLIF-C, Chronic Liver Failure Consortium; INR, international normalized ratio; MELD, model for end-stage liver disease; suPAR, soluble urokinase plasminogen activator receptor.

whether suPAR improved risk stratification, we integrated it with existing models. In patients with AD, adding suPAR consistently increased predictive accuracy (Table S6). For example, MELD-Na improved from AUC 0.759 (95% CI 0.618–0.901) to 0.812 (95% CI 0.679–0.945) when combined with suPAR. Similarly, MELD improved from 0.703 to 0.785, and Child-Pugh score from 0.728 to 0.790.

SuPAR also predicted progression from AD to ACLF. Median suPAR was lower in patients who remained stable (13.4 ng/ml, IQR 10.58–16.1) compared with those who developed ACLF (16.55 ng/ml, IQR 13.45–23.98; *p* = 0.014). ROC analysis identified a cut-off of 14.7 ng/ml (AUC 0.673, 95% CI 0.528–0.812) (Table S5). Regression models further supported suPAR's prognostic role (Table S7). Subgroup analyses showed no significant differences in suPAR levels or outcomes by sex: hospitalization (*p* = 0.370), ICU treatment (*p* = 0.595), or 90-day mortality (*p* = 0.393). Similarly, suPAR did not vary significantly across cirrhosis etiologies. Patients

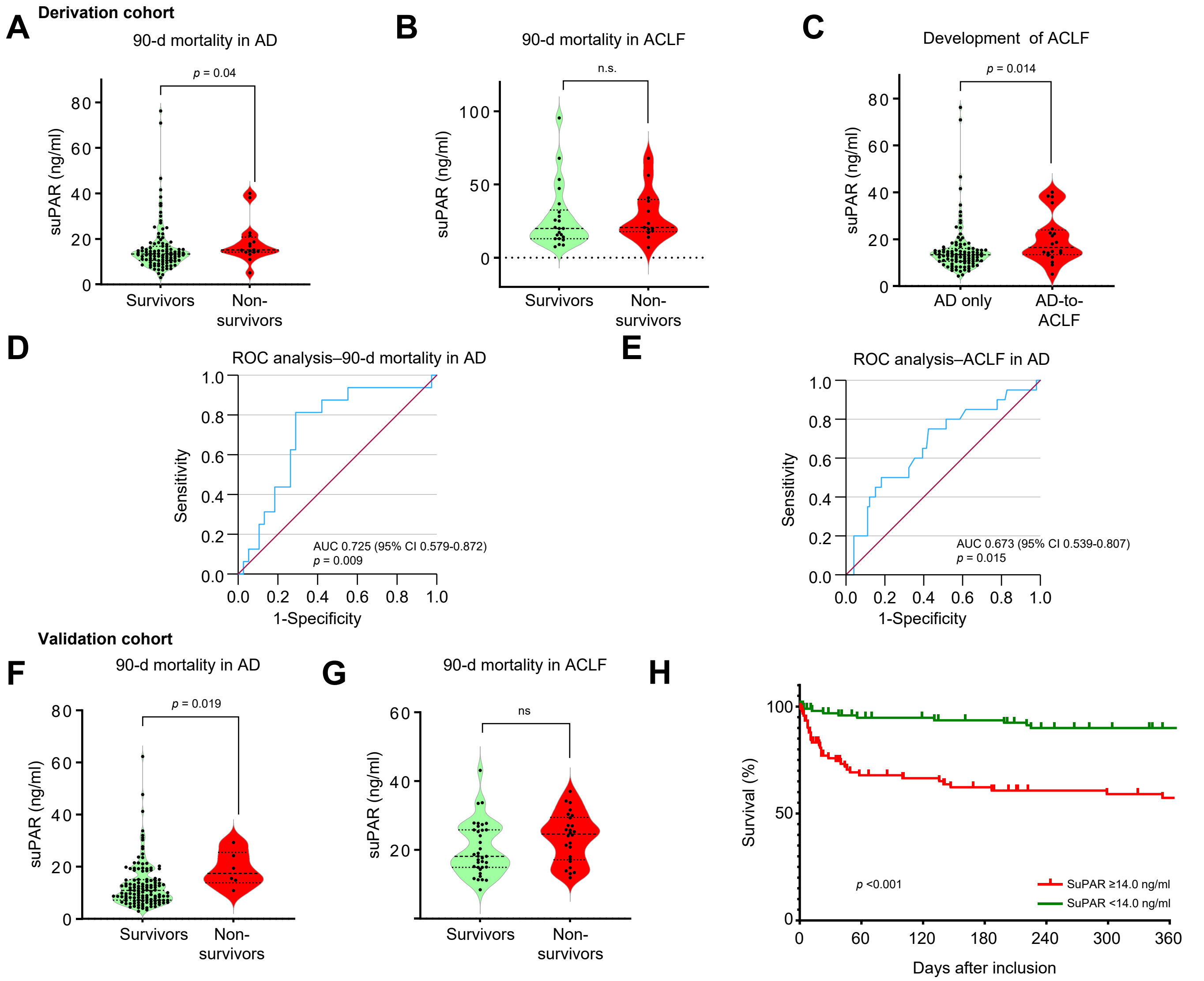

Patients at risk							
suPAR <14.0 ng/ml	103	87	84	80	74	71	67
suPAR ≥14.0 ng/ml	94	51	47	42	36	35	33

Fig. 3. Outcomes in the derivation and validation cohort. (A) In patients with AD (derivation cohort), non-survivors had significantly higher suPAR (median 15.15 ng/ml, IQR 14.20–20.80) compared to survivors/those lost to follow-up (median 13.40 ng/ml, IQR 10.50–16.78; p = 0.039, Mann–Whitney U). (B) In ACLF (derivation cohort), no significant difference was observed between non-survivors (20.70 ng/ml, IQR 17.70–39.80) and survivors/those lost to follow-up (20.00 ng/ml, IQR 12.98–32.60; p = 0.468). (C) Among patients with AD, those who progressed to ACLF had higher suPAR (16.55 ng/ml, IQR 13.45–23.98) than those with stable AD (13.40 ng/ml, IQR 10.58–16.10; p = 0.014). (D) ROC analysis for 90-day mortality in AD showed an AUC of 0.725, with an optimal cut-off of 14.0 ng/ml (sensitivity 0.813, specificity 0.711; p = 0.009). (E) ROC analysis for ACLF development in AD yielded an AUC of 0.673 (p = 0.015). (F) In the validation cohort, AD non-survivors had higher suPAR (15.1 ng/ml, IQR 10.45–25.48) compared to survivors/those lost to follow-up (10.0 ng/ml, IQR 7.05–14.20; p = 0.019). (G) In ACLF (validation cohort), suPAR did not differ significantly between non-survivors (21.4 ng/ml, IQR 14.85–27.05) and survivors/those lost to follow-up (19.0 ng/ml, IQR 15.10–27.15; p = 0.077). (H) Kaplan–Meier survival analysis in AD showed significantly poorer outcomes for patients with suPAR ≥14.0 ng/ml (red) *vs.* <14.0 ng/ml (green). Cox regression: HR = 5.363, 95% CI 2.420–11.886, p <0.001. ACLF, acute-on-chronic liver failure; AD, acute decompensation; suPAR, soluble urokinase plasminogen activator receptor.

with alcohol-related liver disease had comparable suPAR levels (p = 0.086) and outcomes for hospitalization (p = 0.245), ICU treatment (p = 0.220), and 90-day mortality (p = 0.975) compared with other etiologies (Tables S8 and S9).

In summary, elevated suPAR is associated with adverse outcomes and independently predicts prognosis in decompensated cirrhosis. Importantly, combining suPAR with established models improves predictive accuracy for mortality and progression to ACLF, supporting its role as a clinically relevant biomarker.

SuPAR is a mortality marker in decompensated cirrhosis – validation cohort

These findings were corroborated in an independent validation cohort of 135 patients with AD and 62 with ACLF. Most patients were male (71.1%) with a median age of 57 years (IQR 51–65). Alcohol-related liver disease was the predominant etiology, followed by MASLD (metabolic dysfunction-associated steatotic liver disease) and viral hepatitis, though suPAR levels did not differ significantly across etiologies. The

Table 2. Univariate and multivariate analysis for 90 day mortality in patients with decompensated cirrhosis.

	Univariate analysis			Multivariate analysis		
**Baseline factor**	**Exp (B)**	**95% CI Exp (B)**	***p* value**	**Exp (B)**	**95% CI Exp (B)**	***p* value**
Sex (female/male)	0.705	0.245-2.024	0.514			
suPAR cut-off ≥14.0 ng/ml	5.681	1.527-21.140	0.005	5.295	1.377-20.362	0.015
Age (years)	1.204	1.008-1.438	0.041			
Potassium (mmol/L)	3.106	0.434-22.229	0.259			
Sodium (mmol/L)	0.729	0.549-0.970	0.030	0.912	0.832-1.0	0.049
Creatinine (μmol/L)	1.008	0.968-1.049	0.702			
Bilirubin (μmol/L)	1.018	1.002-1.035	0.027			
Albumin (G/L)	0.838	0.687-1.022	0.080			
INR	1.252	1.075-11.378	0.037			
ALT (U/L)	1.000	0.995-1.004	0.935			
ALP (U/L)	1.002	0.992-1.013	0.707			
CRP (mg/L)	1.002	0.986-1.018	0.800			
Hemoglobin (g/L)	1.016	0.947-1.090	0.650			
WBC (1,000/μl)	1.328	1.020-1.729	0.035			
Platelets (1,000/μl)	0.965	0.931-1.000	0.048			
MAP (mmHg)	1.006	0.929-1.089	0.883			

Univariate and multivariate logistic regression for 90-day mortality in decompensated cirrhosis (derivation cohort). Significant univariate variables were entered into a multivariate model with backward elimination. A suPAR cut-off ≥14.0 ng/ml remained an independent predictor of mortality (p = 0.049). ALP, alkaline phosphatase; ALT, alanine aminotransferase; CRP, C-reactive protein; INR, international normalized ratio; MAP, mean arterial pressure; suPAR, soluble urokinase plasminogen activator receptor.

90-day mortality rate was 4.4% for AD and 41.9% for ACLF, lower than in the derivation cohort, likely due to outpatient inclusion in AD and liver transplantation (n = 17). Median plasma suPAR was significantly lower in AD (11 ng/ml, IQR 7.2–15.5) compared with ACLF (21.1 ng/ml, IQR 15.0–27.0; p <0.001). We next examined the prognostic significance of suPAR. In the overall cohort (AD + ACLF), suPAR ≥14.0 ng/ml was associated with increased 28-day mortality (HR 9.589, 95% CI 2.756–33.361, p <0.001) and 90-day mortality (HR 7.899, 95% CI 2.895–21.547, p <0.001).

In subgroup analyses, elevated suPAR predicted 90-day mortality in AD (HR 11.974, 95% CI 1.353–105.939, p = 0.006) but not in ACLF (HR 1.328, 95% CI 0.345–5.109, p = 0.680). Kaplan-Meier analysis confirmed significantly poorer survival for patients with AD and suPAR ≥14.0 ng/ml (Log-rank p <0.001; Fig. 3H). Cox regression yielded a HR of 5.363 (95% CI 2.420–11.886, p <0.001).

Notably, mortality differences were most pronounced within the first 60 days after admission, suggesting that elevated suPAR is particularly predictive of short-term mortality in patients with AD.

Preclinical assessment of suPAR levels in an inflammation-triggered liver disease model

We next investigated whether inflammation, a hallmark of progression from AD to ACLF, drives suPAR release.[7,22] A well-established mouse model of acute-on-chronic liver injury was used, combining CCl_4 injections to induce fibrosis with LPS to trigger systemic inflammation and organ injury[23,24] (Fig. 4A).

Masson's trichrome confirmed bridging fibrosis after CCl_4 exposure (Fig. 4C), while TUNEL staining indicated marked tissue injury following LPS challenge (Fig. 4D).

ELISA showed circulating suPAR levels of 2.34 ng/ml (IQR 1.7–3.1) in controls. Chronic CCl_4 exposure for 10 weeks resulted in similar levels (2.51 ng/ml, IQR 2.39–2.95). However, subsequent LPS injection significantly elevated plasma suPAR (7.77 ng/ml, IQR 4.48–7.95; p = 0.049) (Fig. 4B).

Since suPAR is known to be secreted by circulating immune cell subsets, such as monocytes,[11,12] it is essential to determine the extent to which the liver may serve as an additional source. Immunofluorescence staining for uPAR on FFPE liver slides revealed a significant increase in uPAR expression (p = 0.0037), predominantly localized in non-parenchymal cells. These cells were $CD45^+$ and MPO^+ but negative for CD31, IBA-1, CK-19, Hep Par-1, HNF4α, and CD3, identifying them as neutrophils. In the acute-on-chronic model, $uPAR^+$ cells clustered with inflammatory infiltrates expressing CD45, MPO, IBA-1, and partially CD31 (Fig. 4E).

Single-cell RNA sequencing shows the upregulation of PLAUR in monocytes, macrophages and dendritic cells in decompensated cirrhosis

Next, we next sought to identify the main cell type driving suPAR elevation in cirrhotic livers of patients with cirrhosis. Single-cell transcriptome data was analyzed from a total of seven liver samples of healthy controls and 10 samples from patients with HBV-related cirrhosis.[18–20] After dimensionality reduction using principal component analysis and UMAP (uniform manifold approximation and projection), we identified 23 cell subgroups, which were classified into T cells; myeloid cells; NK cells; B cells and plasma cells (Fig. 5B). *PLAUR*, the gene encoding uPAR, was predominantly expressed in in myeloid cells. Notably, increased *PLAUR* expression was observed in subpopulations of monocytes, macrophages, and dendritic cells in patients with HBV-related cirrhosis compared to healthy controls. In dendritic cells, cirrhosis was associated with increased *PLAUR* expression in the conventional type 1 and 2 dendritic cell subpopulations, while no such increase was observed in the plasmacytoid dendritic cell subpopulation. Monocytes exhibited elevated *PLAUR* expression across all clusters. In macrophages, *PLAUR* upregulation was identified in specific subclusters characterized by the expression of markers such as C1QA, CCL5, CD5L, CD9/TL18, CD9/TL18BP, CXCL10, and FCN1 (Fig. 5D). However, this specific uPAR expression pattern may be associated with chronic viral hepatitis and could differ in other etiologies of cirrhosis. A KEGG analysis suggests that *PLAUR* is involved in various functions, primarily related to immune responses and autoimmune diseases within these subclusters (Fig. S1). However, the exact function remains speculative.

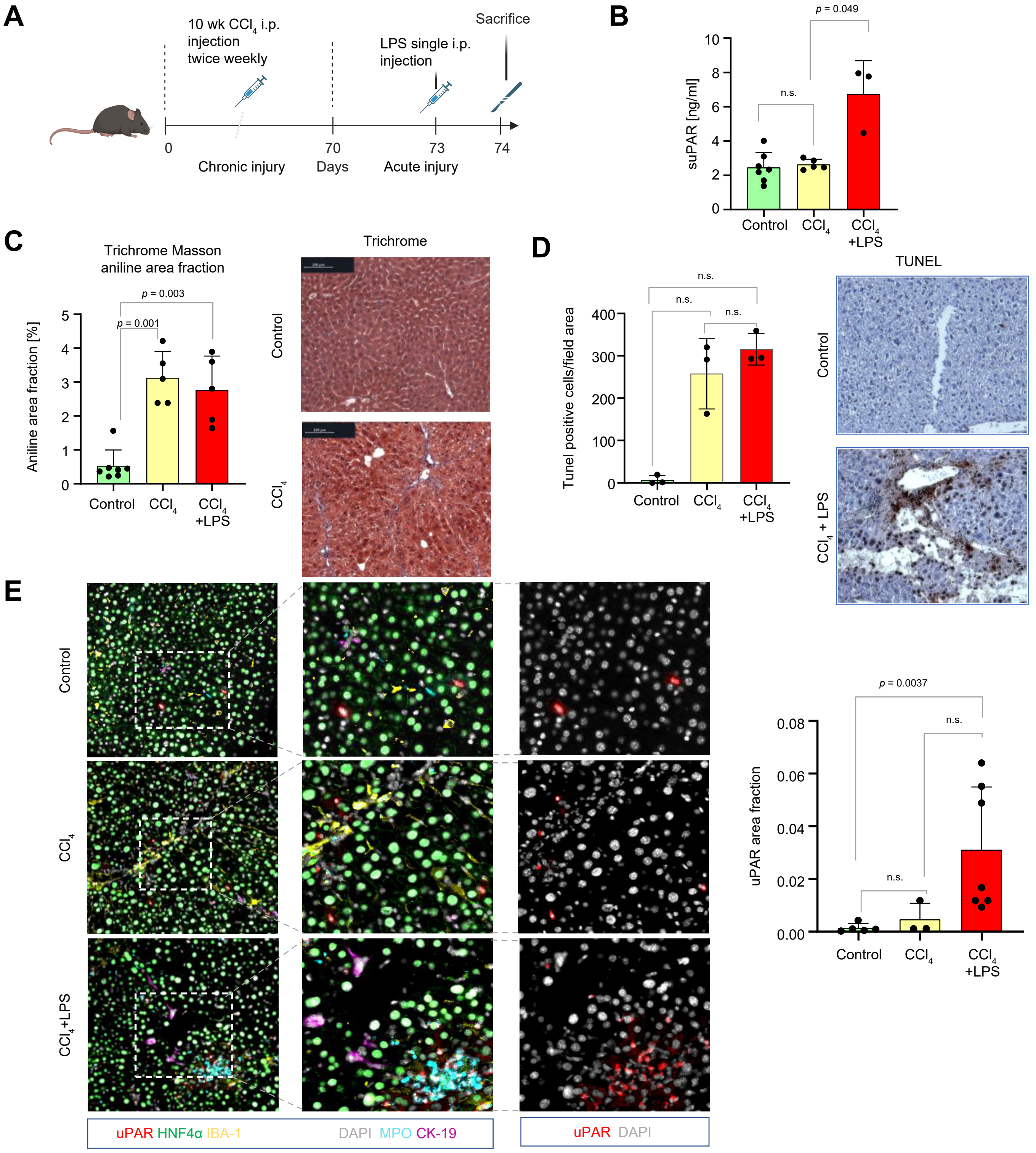

Fig. 4. SuPAR in murine inflammation triggered acute-on-chronic liver injury model. (A) C57BL/6J mice (8–10 weeks) received i.p. CCl_4 for 10 weeks, followed by i.p. LPS after 3 days and were sacrificed 24 h later. (B) Plasma suPAR levels (ng/ml) were significantly higher after LPS challenge (CCl_4 + LPS) compared to CCl_4 alone (p = 0.049, Kruskal–Wallis with Bonferroni correction). (C) Masson's trichrome staining (20 ×) of control, CCl_4, and CCl_4 + LPS livers showed fibrosis (violet connective tissue, red cytoplasm, blue nuclei). Fibrosis area fraction was significantly increased in CCl_4 (p = 0.001) and CCl_4 + LPS (p = 0.003) *vs.* controls (Kruskal–Wallis with Bonferroni correction) (D) TUNEL HRP-DAB staining (20 ×) revealed apoptotic nuclei (brown) with hematoxylin counterstain (blue). The apoptotic fraction was significantly higher in CCl_4 + LPS *vs.* CCl_4 and controls (p = 0.025) (Kruskal–Wallis with Bonferroni correction) . (E) Multiplex immunofluorescence (uPAR, red; DAPI, grey) demonstrated increased uPAR expression in CCl_4 + LPS *vs.* controls (p = 0.0037). CCl_4, carbon tetrachloride; LPS, lipopolysaccharide; suPAR, soluble urokinase plasminogen activator receptor; uPAR, urokinase plasminogen activator receptor.

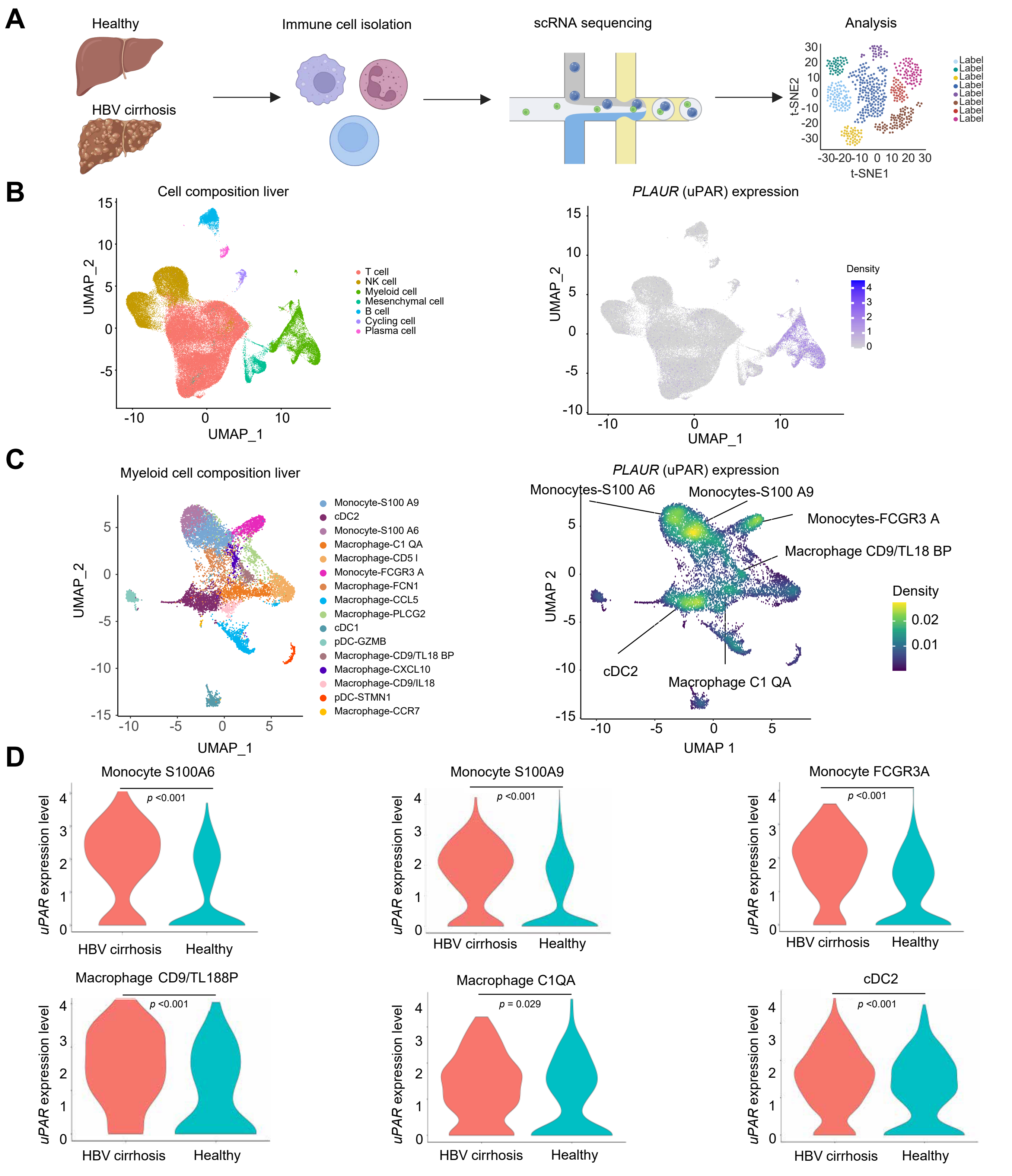

Fig. 5. Single-cell RNA sequencing in liver immune cells from HBV-related cirrhosis compared to healthy controls. (A) CD45^{+} liver cells from patients with HBV-cirrhosis and healthy controls were re-analysed for uPAR expression. (B) UMAP dimensionality reduction for principal component analysis and *PLAUR* expression density in liver cells indicating *PLAUR* expression mostly in myeloid cells. (C) Within myeloid cells, PLAUR was enriched in monocytes, dendritic cells, and macrophages. (D) Subcluster analysis showed elevated *PLAUR* in cirrhosis *vs.* controls: monocytes (S100A6, p <0.001; S100A9, p <0.001; FCGR3A, p <0.001), macrophages (CD9/TL188P, p <0.001; C1QA, p = 0.029), and dendritic cells (cDC2, p <0.001) (Mann–Whitney *U*). cDC1/2, conventional type 1/2 dendritic cell; pDC, plasmacytoid dendritic cell; uPAR, urokinase plasminogen activator receptor; UMAP, uniform manifold approximation and projection.

Discussion

AD and ACLF are progressive diseases with high mortality and limited treatment options.[25] Systemic and hepatic inflammation are key drivers of disease progression.[7,24,26] Consequently, there is an urgent need for reliable biomarkers predicting prognosis and guiding diagnostic and therapeutic strategies.

Our dataset, which includes healthy controls as well as patients with CC, AD, and ACLF, provides compelling evidence supporting suPAR as a biomarker of liver disease severity. This conclusion, drawn from both derivation and validation cohorts, preclinical murine models, and human single-cell RNA sequencing data, further establishes suPAR's relevance in predicting disease progression and outcomes in cirrhosis.

Our study highlights that circulating suPAR levels closely correlate with the worsening of cirrhosis. It also identifies a suPAR cut-off of 14.0 ng/ml as a significant risk factor for this progression, showing high sensitivity and specificity in predicting 90-day mortality. Remarkably, this predictive accuracy surpasses that of the MELD score within the studied cohort. Importantly, incorporating suPAR into established predictive models enhanced their ability to predict patient outcomes. This suggests suPAR-based predictive models could serve as valuable alternatives to traditional scoring systems. Though not powered for new predictive models, this study supports larger biomarker trials to validate and integrate suPAR into predictive frameworks. We identified a cut-off consistent with previous findings, such as those in patients with HBV-induced ACLF, where a suPAR cut-off of 14.7 ng/ml was identified as an independent risk factor for disease progression,[27] which strengthens the argument for suPAR as a reliable biomarker across different etiologies of liver disease.

Our data align with previous studies which explored the prognostic role of suPAR in end-stage liver disease. For instance, Gaenaes *et al.*[28] enrolled 105 patients with cirrhosis and 19 healthy controls, demonstrating that suPAR levels correlated with disease severity, particularly in those with a Child-Pugh C score, ascites, and elevated bilirubin levels. Another prospective cohort study involving 159 patients with CLD, including 98 with cirrhosis, also found suPAR to predict mortality or the need for liver transplantation, with a suggested cut-off of 9 ng/ml[11]. Similarly, Loosen *et al.* demonstrated that suPAR could predict mortality following TIPS (transjugular intrahepatic portosystemic shunt) implantation.[29] Moreover, the differential regulation of suPAR levels in various compartments, such as ascites and blood, provides crucial insights into the potential progression of liver disease.[12]

Several studies have demonstrated that patients with severe fibrosis exhibit significantly higher levels of suPAR and IL-10 than those with mild fibrosis.[30–32] Interestingly, in our cohorts, the similar suPAR levels in healthy controls and patients with CC suggest that suPAR reflects an inflammatory environment rather than indicating liver fibrosis. This hypothesis was further confirmed in a preclinical model of inflammation-driven acute-on-chronic liver injury, where the injection of LPS on top of CCl_4-induced liver fibrosis resulted in elevated suPAR levels. Thus, increased circulating suPAR levels seem to reflect immune cell activation and systemic inflammation, consistent with current pathomechanistic paradigms.[22]

Recent studies by Loosen *et al.* found significantly higher suPAR levels in hepatic venous blood than in portal venous blood, suggesting that the injured liver itself may be a key source of suPAR.[29] This finding led us to focus on suPAR expression within the liver. In our animal model, uPAR was predominantly expressed by non-epithelial cells. Further analysis of single-cell RNA sequencing datasets identified that uPAR expression in hepatic leukocytes was primarily observed in myeloid subsets such as S100A6-monocyte and CCL5-macrophage subsets, suggesting a unique role for uPAR in innate immune responses during AD and ACLF, while the exact function remains speculative.

UPAR is expressed in multiple cell types and linked to various diseases. Halm *et al.* identified bone marrow-derived immature myeloid cells as a source of suPAR in proteinuric kidney diseases, while monocytes and macrophages were found to contribute to suPAR levels in sepsis.[33] In cardiovascular diseases, endothelial cells and macrophages play a role in suPAR elevation,[34] while in cancer, tumor cells and cancer-associated fibroblasts are key contributors.[35] Furthermore, in ascites, monocytes, neutrophils, and CD14+ peritoneal macrophages have been shown to express suPAR.[12] Other studies reported that hepatic leukocyte uPAR expression appears predominantly in proinflammatory monocytes and unconventional lymphocyte subsets, emphasizing its importance in innate immunity.[10] However, the specific cellular source of suPAR in cirrhosis remains to be fully elucidated. Elevated suPAR levels occur in many diseases, highlighting the need to define its prognostic value specifically in liver disease. The extent to which circulating immune cells, such as neutrophils,[9] and other tissue-resident immune cells from various organs contribute to circulating suPAR levels remains unclear. Due to the limited availability of multi-organ samples from patients with AD and ACLF, it may be challenging to obtain sufficient human data from other organs to explore this further. Notably, our data show no significant difference in suPAR levels between patients with and without acute infections, suggesting that suPAR reflects liver disease-related immune activation rather than infection.

A key limitation is the descriptive nature of our results, which do not establish causality. Additionally, the etiologies of cirrhosis and the precipitating events of AD and ACLF varied across our cohorts. In particular, HBV (n = 8) was a rare cause of cirrhosis in the derivation cohort, which limits direct comparability with experimental models. UPAR expression and the associated inflammation pattern may be related to the underlying etiology of cirrhosis and could differ across various etiological subtypes. However, consistency with previous studies and preclinical data emphasizes a consistent and robust effect across a heterogeneous population and highlights the potential relevance of suPAR in AD and ACLF. The identified suPAR cut-off could serve as a useful predictive tool prior to the application of the CLIF-C AD score, a well-established method for assessing the risk of progression from AD to ACLF.[36] Nevertheless, our retrospective analysis revealed that suPAR levels could not predict mortality in the ACLF group at admission, likely reflecting the complex interplay of multiple factors influencing patient outcomes. This observation may also be due to the relatively small number of patients with ACLF in our cohort. While suPAR did not

outperform the CLIF-C AD score, it provided an additional layer of stratification for cirrhosis, particularly in predicting the transition from AD to ACLF.

Our study highlights the predictive potential of circulating suPAR levels in patients with AD and ACLF, particularly in identifying those at risk of progression from AD to ACLF. These findings underscore inflammation-related biomarkers not only in reflecting distinct pathophysiological mechanisms but also in suggesting inflammation as a potential therapeutic target, as indicated in previous studies.[23,24] Recent studies have also identified liver-resident immune subsets as a potential source of suPAR. Integrating suPAR into multimodal predictive models could enhance the ability to identify patients at risk of disease progression.

Affiliations

[1]*Charité – Universitätsmedizin Berlin, corporate member of Freie Universität Berlin and Humboldt-Universität zu Berlin, Department of Hepatology & Gastroenterology, Campus Virchow-Klinikum and Campus Charité Mitte, Berlin, Germany;* [2]*Charité – Universitätsmedizin Berlin, corporate member of Freie Universität Berlin and Humboldt-Universität zu Berlin, Institute of Diagnostic Laboratory Medicine, Clinical Chemistry and Pathobiochemistry, Berlin, Germany;* [3]*Labor Berlin - Charité Vivantes GmbH, Berlin, Germany;* [4]*Cancer Research Center, School of Medicine, Xiamen University, Xiamen, China;* [5]*European Foundation for the Study of Chronic Liver Failure, Barcelona, Spain;* [6]*Liver Failure Group, Institute for Liver and Digestive Health, University College London Medical School, Royal Free Hospital, London, United Kingdom;* [7]*Leipzig University Medical center, Division of Hepatology, Department of Medicine II, Leipzig, Germany;* [8]*3rd Department of Medicine, University Hospital Augsburg, Augsburg, Germany*

Abbreviations

ACLF, acute-on-chronic liver failure; AD, acute decompensation; CC, compensated cirrhosis; CCl_4, carbon tetrachloride; CLD, chronic liver disease; CRP, C-reactive protein; HR, hazard ratio; ICU, intensive care unit; IL-interleukin-; LPS, lipopolysaccharide; MELD, model for end-stage liver disease; suPAR, soluble urokinase plasminogen activator receptor; uPAR, urokinase plasminogen activator receptor.

Financial support

This study was funded by institutional funding. CE was part funded by the Else-Kroener-Fresenius Foundation (Excellence Funding). PK was funded by Else-Kroener-Fresenius Foundation (First and Second Application program).

Conflicts of Interest

CE has received advisory fees, travel reimbursement and lecture fees from Boehringer Ingelheim, Albireo/Ipsen and Gilead. CE is a shareholder of Hepyx Ltd and is listed as an inventor for the treatment of liver failure with stem-cell mobilisation and toll-like receptor 4 antagonists which is licensed to Hepyx Ltd. TB has received grants/research supports from Abbvie, Advance, Gilead, Humedics, Intercept, Norgine, Orphalan, Sequana Medical and has received of honoraria or consultation fees/advisory board from Abbvie, Alexion, Albireo, Bayer, Gilead, GSK, Eisai, Humedics, Intercept, Ipsen, MSD/Merck, Novartis, Orphalan, Sequana Medical. TB participates in the company sponsored speaker's bureau of Abbvie, Advance Pharma, Alexion, Albireo, Bayer, Gilead, Eisai, Falk Foundation, Intercept, Ipsen, MedUpdate GmbH, MSD/Merck, Orphalan, Sequana Medica. RJ has research collaborations with Yaqrit. He is the founder of Yaqrit limited, a spin out company from University College London. He has also co-founded Hepyx Ltd. and Cyberliver Ltd. CdlPR is currently employed by AstraZeneca; however, this work was conducted while affiliated with the European Foundation for the Study of Chronic Liver Failure Barcelona. MH is currently employed by Boehringer Ingelheim Pharma GmbH & Co; however, this work was conducted while affiliated with the Charité – Universitätsmedizin Berlin, Department of Hepatology & Gastroenterology, Campus Virchow-Klinikum and Campus Charité Mitte. FT has received honoraria for consulting or lectures from AstraZeneca, Gilead, AbbVie, BMS, Boehringer, Madrigal, Intercept, Falk, Inventiva, MSD, GSK, Orphalan, Merz, Pfizer, Alnylam, CSL Behring, Novo Nordisk, Sanofi, and Novartis. FT's laboratory has received research funding from Gilead, AstraZeneca, Agomab, and MSD (funding to the institution).
Please refer to the accompanying ICMJE disclosure forms for further details.

Authors contributions

Conceptualization (CE, SL), Methodology (CE, SL, MH), Investigation and data curation (SL, KK, RM, MD, FA, RV, JS, NA, JF, RM, TB, FA, RS, JW, QB, TH, ZZ, NW), Writing – original draft (SL, PK), Review & editing (SL, PK, CE, QB, TH, KK, RJ, FT, NW, TB), Formal Analysis (SL, CE, HB, CdlPR, NW), and Supervision (HB, CE, FT, PK) Funding acquisition (CE). All authors have read and agreed to the published version of the manuscript.

Data availability

Further supporting information is available in the supplementary materials, and additional data can be obtained from the authors upon reasonable request.

Ethics approval

All animal experiments complied with ethical regulations and were approved by the committee on Research Animal Care of the Charité—Universitätsmedizin Berlin, Berlin, Germany (License number: G0174/20). Human tissue samples for scRNA sequencing were collected under approval from Ethics Review Center of the First Affiliated Hospital of Harbin Medical University (License number: 20211179) as well as data from two distinct studies (License numbers: HRA001730; HRA000069) as stated previously.[1] Human samples were collected and corresponding medical data was retrieved from the DASIMAR trial (ClinicalTrials.gov Identifier: NCT01071746) under approval by the joint UCL/UCLH Committees on the Ethics of Human Research (Committee A), with Research Ethics Committee reference number 08/H0714/8. Human samples were collected and corresponding medical data was retrieved under approval from the Ethics committee of the Faculty of Medicine of the Leipzig University (License number: 240/20-ek).

Acknowledgments

We are thankful for technical support from the Department of Hepatology and Gastroenterology, Charité - Universitätsmedizin Berlin (CVK) and for statistical support from the Institute of Biometry and Clinical Epidemiology, Charité - Universitätsmedizin Berlin. CE was part funded by the Else Kroener-Fresenius-Stiftung.

Supplementary data

Supplementary data to this article can be found online at https://doi.org/10.1016/j.jhepr.2025.101677.

References

Author names in bold designate shared co-first authorship

[1] Bai Q, Li R, He X, et al. Single-cell landscape of immune cells during the progression from HBV infection to HBV cirrhosis and HBV-associated hepatocellular carcinoma. Front Immunol 2023;14:1320414.

[2] Huang DQ, Terrault NA, Tacke F, et al. Global epidemiology of cirrhosis - aetiology, trends and predictions. Nat Rev Gastroenterol Hepatol 2023;20(6):388–398.

[3] **Lamatsch S**, **Sittner R**, Tacke F, et al. Novel drug discovery strategies for the treatment of decompensated cirrhosis. Expert Opin Drug Discov 2022;17(3):273–282. https://doi.org/10.1080/17460441.2022.2020755.

[4] **Juanola A**, **Ma AT**, De Wit K, et al. Novel prognostic biomarkers in decompensated cirrhosis: a systematic review and meta-analysis. Gut 2023;73(1):156–165. https://doi.org/10.1136/GUTJNL-2023-329923.

[5] Higazi AA, El-Haj M, Melhem A, et al. Immunomodulatory effects of plasminogen activators on hepatic fibrogenesis. Clin Exp Immunol 2008;152(1):163–173.

[6] **Amor C**, **Feucht J**, Leibold J, et al. Senolytic CAR T cells reverse senescence-associated pathologies. Nat 2020;583(7814):127–132. https://doi.org/10.1038/s41586-020-2403-9. 2020 5837814.
[7] Engelmann C, Zhang IW, Clària J. Mechanisms of immunity in acutely decompensated cirrhosis and acute-on-chronic liver failure. Liver Int. 2025 Mar;45(3):e15644. https://doi.org/10.1111/liv.15644. Epub 2023 Jun 27.
[8] Albillos A, Martin-Mateos R, Van der Merwe S, et al. Cirrhosis-associated immune dysfunction. Nat Rev Gastroenterol Hepatol 2022;19(2):112–134.
[9] Gussen H, Hohlstein P, Bartneck M, et al. Neutrophils are a main source of circulating suPAR predicting outcome in critical illness. J Intensive Care 2019;7(1).
[10] **Koch A**, **Zimmermann HW**, Gassler N, et al. Clinical relevance and cellular source of elevated soluble urokinase plasminogen activator receptor (suPAR) in acute liver failure. Liver Int Off J Int Assoc Study Liver 2014;34(9):1330–1339. https://doi.org/10.1111/LIV.12512.
[11] **Zimmermann HW**, **Koch A**, Seidler S, et al. Circulating soluble urokinase plasminogen activator is elevated in patients with chronic liver disease, discriminates stage and aetiology of cirrhosis and predicts prognosis. Liver Int Off J Int Assoc Study Liver 2012;32(3):500–509. https://doi.org/10.1111/J.1478-3231.2011.02665.X.
[12] Zimmermann HW, Reuken PA, Koch A, et al. Soluble urokinase plasminogen activator receptor is compartmentally regulated in decompensated cirrhosis and indicates immune activation and short-term mortality. J Intern Med 2013;274(1):86–100.
[13] **Huang Z**, **Wang N**, Huang S, et al. Increased Serum Soluble Urokinase Plasminogen Activator Receptor Predicts Short-Term Outcome in Patients with Hepatitis B-Related Acute-on-Chronic Liver Failure. Gastroenterol Res Pract 2019;2019(1):3467690. https://doi.org/10.1155/2019/3467690.
[14] Moreau R, Jalan R, Gines P, et al. Acute-on-chronic liver failure is a distinct syndrome that develops in patients with acute decompensation of cirrhosis. Gastroenterology 2013;144(7):1426–1437. 1437.e1-9.
[15] WMA - the world medical association-WMA declaration of Helsinki – ethical principles for medical research involving human subjects. Accessed August 8, 2023. https://www.wma.net/policies-post/wma-declaration-of-helsinki-ethical-principles-for-medical-research-involving-human-subjects/.
[16] Percie du Sert N, Hurst V, Ahluwalia A, et al. The ARRIVE guidelines 2.0: updated guidelines for reporting animal research. Plos Biol 2020;18(7):e3000410.
[17] Guillot A, Kohlhepp MS, Bruneau A, et al. Deciphering the immune microenvironment on A single archival formalin-fixed paraffin-embedded tissue section by an immediately implementable multiplex fluorescence immunostaining protocol. Cancers 2020;12(9):2449.
[18] **Bai Q**, **Hong X**, **Lin H**, et al. Single-cell landscape of immune cells in human livers affected by HBV-related cirrhosis. JHEP Rep Innov Hepatol 2023;5(11):100883. https://doi.org/10.1016/j.jhepr.2023.100883.
[19] Ramachandran P, Dobie R, Wilson-Kanamori JR, et al. Resolving the fibrotic niche of human liver cirrhosis at single-cell level. Nature 2019;575(7783):512–518.
[20] Zhang Q, He Y, Luo N, et al. Landscape and dynamics of single immune cells in hepatocellular carcinoma. Cell 2019;179(4):829–845.e20.
[21] **Stuart T**, **Butler A**, Hoffman P, et al. Comprehensive Integration of Single-Cell Data. Cell 2019;177(7):1888–1902.e21. https://doi.org/10.1016/j.cell.2019.05.031.
[22] Engelmann C, Clària J, Szabo G, et al. Pathophysiology of decompensated cirrhosis: portal hypertension, circulatory dysfunction, inflammation, metabolism and mitochondrial dysfunction. J Hepatol 2021;75:S49–S66.
[23] Engelmann C, Habtesion A, Hassan M, et al. Combination of G-CSF and a TLR4 inhibitor reduce inflammation and promote regeneration in a mouse model of ACLF. J Hepatol 2022;77(5):1325–1338.
[24] **Engelmann C**, **Sheikh M**, Sharma S, et al. Toll-like receptor 4 is a therapeutic target for prevention and treatment of liver failure. J Hepatol 2020;73(1):102–112. https://doi.org/10.1016/j.jhep.2020.01.011.
[25] **Engelmann C**, **Thomsen KL**, Zakeri N, et al. Validation of CLIF-C ACLF score to define a threshold for futility of intensive care support for patients with acute-on-chronic liver failure. Crit Care Lond Engl 2018;22(1):254. https://doi.org/10.1186/s13054-018-2156-0.
[26] Baloda V, Anand A, Yadav R, et al. Histologic changes in core-needle liver biopsies from patients with acute-on-chronic liver failure and independent histologic predictors of 28-day mortality. Arch Pathol Lab Med 2022;146(7):846–854.
[27] **Wang Y**, **Wu F**, **Chen C**, et al. Soluble urokinase plasminogen activator receptor is associated with short-term mortality and enhanced reactive oxygen species production in acute-on-chronic liver failure. BMC Gastroenterol 2021;21(1):429. https://doi.org/10.1186/s12876-021-02006-x.
[28] Garnæs E, Mortensen C, Hobolth L, et al. Kinetics of the soluble urokinase plasminogen activator receptor (suPAR) in cirrhosis. PLoS One 2019;14(8):e0220697.
[29] **Loosen SH**, **Benz F**, **Mohr R**, et al. Soluble urokinase plasminogen activator receptor levels predict survival in patients with portal hypertension undergoing TIPS. JHEP Rep Innov Hepatol 2024;6(5):101054. https://doi.org/10.1016/j.jhepr.2024.101054.
[30] Sevgi DY, Bayraktar B, Gündüz A, et al. Serum soluble urokinase-type plasminogen activator receptor and interferon-γ-induced protein 10 levels correlate with significant fibrosis in chronic hepatitis B. Wien Klin Wochenschr 2016;128(1–2):28–33.
[31] Özdirik B, Maibier M, Scherf M, et al. Soluble urokinase plasminogen activator receptor levels are associated with severity of fibrosis in patients with primary sclerosing cholangitis. J Clin Med 2022;11(9):2479.
[32] Berres ML, Schlosser B, Berg T, et al. Soluble urokinase plasminogen activator receptor is associated with progressive liver fibrosis in hepatitis C infection. J Clin Gastroenterol 2012;46(4):334–338.
[33] Nusshag C, Wei C, Hahm E, et al. suPAR links a dysregulated immune response to tissue inflammation and sepsis-induced acute kidney injury. JCI Insight 2023;8(7):e165740.
[34] **Hindy G**, **Tyrrell DJ**, **Vasbinder A**, et al. Increased soluble urokinase plasminogen activator levels modulate monocyte function to promote atherosclerosis. J Clin Invest 2022;132(24):e158788. https://doi.org/10.1172/JCI158788.
[35] **Tian B**, **Chen X**, **Zhang H**, et al. Urokinase plasminogen activator secreted by cancer-associated fibroblasts induces tumor progression via PI3K/AKT and ERK signaling in esophageal squamous cell carcinoma. Oncotarget 2017;8(26):42300–42313. https://doi.org/10.18632/oncotarget.15857.
[36] **Trebicka J**, **Fernandez J**, Papp M, et al. PREDICT identifies precipitating events associated with the clinical course of acutely decompensated cirrhosis. J Hepatol 2021;74(5):1097–1108. https://doi.org/10.1016/j.jhep.2020.11.019.

Keywords: Chronic liver disease; suPAR; Biomarker; Cirrhosis; ACLF; PLAUR.
Received 1 March 2025; received in revised form 2 November 2025; accepted 5 November 2025; Available online 11 November 2025

Journal of Hepatology, Volume *8*

Supplemental information

Soluble urokinase plasminogen activator receptor is a prognostic biomarker in decompensated cirrhosis

Sven Lamatsch, Mohsin Hassan, Kai Kappert, Hilmar Berger, Qingquan Bai, Zhengyang Zhao, Nirbaanjot Walia, Carlos De La Peña-Ramirez, Raphael Mohr, Münevver Demir, Juan Wang, Fabian Artusa, Richard Sittner, Fausto Andreola, Rhea Veelken, Florian van Boemmel, Jonas Schumacher, Niklas Aehling, Janett Fischer, Rajeshwar Mookerjee, Tianhui Hu, Thomas Berg, Rajiv Jalan, Frank Tacke, Pavitra Kumar, and Cornelius Engelmann

Soluble urokinase plasminogen activator receptor is a prognostic biomarker in decompensated cirrhosis

Sven Lamatsch, Mohsin Hassan, Kai Kappert, Hilmar Berger, Qingquan Bai, Zhengyang Zhao, Nirbaanjot Walia, Carlos De La Peña-Ramirez, Raphael Mohr, Münevver Demir, Juan Wang, Fabian Artusa, Richard Sittner, Fausto Andreola, Rhea Veelken, Florian van Boemmel, Jonas Schumacher, Niklas Aehling, Janett Fischer, Rajeshwar Mookerjee, Tianhui Hu, Thomas Berg, Rajiv Jalan, Frank Tacke, Pavitra Kumar, Cornelius Engelmann

Table of contents

Supplementary Text……………………………………………………………………2

Table S1…………………………………………………………………………………...9

Table S2…………………………………………………………………………………10

Table S3…………………………………………………………………………………11

Table S4…………………………………………………………………………………15

Table S5…………………………………………………………………………………16

Table S6…………………………………………………………………………………17

Table S7…………………………………………………………………………………18

Table S8…………………………………………………………………………………19

Table S9…………………………………………………………………………………22

Fig. S1…………………………………………………………………………………...26

Supplementary references……………………………………………………………..27

Supplementary Text 1: Methods in detail

2.1 Study Design

In a retrospective study, circulating blood suPAR concentrations were assessed in plasma samples from patients with liver cirrhosis across two independent cohorts. The derivation cohort (n=178) consisted of healthy controls (n=6) and patients with CC (n=17), AD (n=120) liver cirrhosis, and ACLF (n=35). The validation cohort (n=197) included patients with AD (n=135) or ACLF (n=62). Plasma samples were collected upon hospital admission, with clinical data retrieved from medical records. The primary study endpoint was survival after 90 days. AD was defined as the occurrence of one or more major liver disease complications, including gastrointestinal bleeding, ascites, HE, and hepatorenal syndrome (HRS). ACLF was defined according to EASL-CLIF ACLF criteria, indicating acute decompensation of preexisting liver cirrhosis and organ failure[1]. The study adhered to good clinical practice principles and the Declaration of Helsinki (1951), with informed written consent obtained from all participants or their legal representatives before study inclusion[2].

2.2 Participating centers and patient cohort

Plasma samples and clinical data for the derivation cohort were sourced from patients hospitalized at the University College London Hospitals with acute decompensation of cirrhosis, as part of the prospective, observational DASIMAR study (Registration number: NCT01071746). This included patients with decompensated liver cirrhosis of any aetiology. The study aimed to identify biomarkers of AD and ACLF. Exclusions were made for patients with malignancies (including hepatocellular carcinoma), and individuals who had undergone major surgery, had unsolved surgical issues or were pregnant. Since the indication of

orthotopic liver transplantation (OLT) could not be determined retrospectively and the number of patients with OLT was low (n=4), all patients who underwent OLT within 90 days of the follow-up period were excluded from the analysis. For the validation cohort plasma samples and corresponding clinical data were obtained from patients with liver cirrhosis who were treated at the Leipzig University Hospital, including both outpatients and inpatients, also including patients who underwent liver transplantation and were analysed retrospectively. The presence of cirrhosis was determined by histopathological workup or non-invasive assessment of liver stiffness and typical findings on imaging, laboratory values, and medical history.

2.3 Measurement of circulating suPAR levels

Blood samples underwent centrifugation at 4,500g for 10 minutes. Heparin plasma was promptly frozen at -80° C and stored until analysis. SuPAR plasma levels were assessed using the suPARnostic® TurbiLatex (Nr. T004, suPARnostic, ViroGates, Birkerød, Denmark) on a clinical chemistry analyzer (Roche Diagnostics) at Labor Berlin – Charité Vivantes GmbH, laboratory of Charité – Universitätsmedizin Berlin, Germany. Measuring range of the test is 1.8 ng/ml to 16.0 ng/ml with a limit of detection of 1.2 ng/ml and a repeatability coefficient of variation (CV) of < 4.0% (intra-assay precision), and a CV between days of < 10% (inter-assay precision) (manufacturer performance data). Standard laboratory parameters (e.g., hemoglobin, white blood cell count (WBC), platelets, total bilirubin, creatinine, Alanine aminotransferase (ALT), Alkaline phosphatase (ALP), C-reactive protein (CRP), albumin, and International normalized ratio (INR)) were measured at the central laboratory institutions of participating centers and retrieved from patients' electronic medical records.

2.4 Mouse chronic liver disease models

All experimental protocols were conducted in compliance with local regulatory authorities' oversight and approval (approval-no: G-0174/20). Results were reported following the guidelines outlined in the ARRIVE guidelines[3]. During experiments, animals were closely monitored, and if there was a rapid deterioration in their condition, they were humanely euthanized. Euthanasia for all experimental groups was performed via exsanguination through the inferior vena cava (IVC) under general anesthesia induced by isoflurane (cp-pharma, Germany). C57B6/J mice, aged 8-10 weeks and weighing between 20 to 40 g, were obtained from the central animal facility Forschungseinrichtungen für Experimentelle Medizin (FEM) at Charité – Universitätsmedizin Berlin. Animals were randomly assigned by investigators to different intervention or control groups and were provided a standard chow diet and water. In an inflammation-triggered model, animals received intraperitoneal (i.p.) injections of 0.6 ml/kg BW CCl4 diluted in corn oil (MERCK, Germany) twice a week for 10 weeks. Three days after the final CCl4 injection, animals received an i.p. injection of 2 mg/kg BW LPS (Sigma-Aldrich, USA) derived from Klebsiella pneumonia. Animals were euthanized 24 hours after the LPS injection.

2.5 Masson's trichrome staining

Masson's trichrome staining was conducted on formalin-fixed paraffin-embedded (FFPE) 4 μm mouse liver tissue sections. The Trichrome Stain Kit (Connective Tissue Stain) from Abcam (Catalog #: ab150686) was utilized following the manufacturer's instructions. To quantify connective tissue, fluorescence microscopy imaging was performed using a ZEISS OBSERVER 7 with Colibri 7 illumination at 631/33 nm, and detection by Axiocam712 mono 12MP (4248x2832 pixel) with a pixel size of 3.1x3.1 μm, using a 20xPlnApo NA0.8 objective. Analysis of images was

conducted by measuring area fraction in FIJI 1.53o. Brightfield microscopy was conducted using a ZEISS OBSERVER 7 with Colibri 7 brightfield illumination, detection by Axiocam305 color 5MP (2464x2056 pixel) with a pixel size of 3.45x3.45 µm, using a 20xLDPln NA0.4 objective.

2.6 TUNEL staining

TUNEL staining was conducted on FFPE 4 µm mouse liver tissue sections. The in Situ Cell Death Detection Kit, POD from Roche (Catalog #: 11684817910) was utilized following the manufacturer's instructions. Brightfield microscopy was conducted using a ZEISS OBSERVER 7 with Colibri 7 brightfield illumination, detection by Axiocam305 color 5MP (2464x2056 pixel) with a pixel size of 3.45x3.45 µm, using a 20xLDPln NA0.4 objective.

2.7 Multiplex Immune Fluorescence Microscopy

Multiplex immune staining, involving repetitive immunostaining and antibody stripping, was conducted on FFPE 4 µm mouse liver tissue sections following the established facility protocol[4]. Deparaffinization and rehydration were achieved using xylene (Roth, Germany) and ethanol (Roth, Germany), respectively, with antigen retrieval performed using Tris-EDTA (pH=9). Sections underwent one-hour blocking in PBS containing 2% normal goat serum, followed by overnight incubation with primary antibodies. Staining was achieved using fluorescent dye-conjugated secondary antibodies. Details of the primary and secondary antibodies used are provided in Supplementary Table 1 and 2. Nuclear staining was performed using 4′,6-diamidino-2-phenylindole (DAPI). Antibody stripping was executed using 2-mercaptoethanol/SDS. Excitation fluorescence microscopy imaging was conducted using a ZEISS OBSERVER 7 with Colibri 7 illumination at various wavelengths, with detection by Axiocam712 mono 12MP (4248x2832 pixel) and a pixel size of 3.1x3.1

µm, using a 20xPlnApo NA0.8 objective. Image analysis, including area fraction determination, was carried out using FIJI 1.54p. For multiplex analysis, scans were aligned, hyperstacked, and concatenated using the FIJI HyperStackReg V5.6 plugin.

2.8 suPAR measurement by ELISA in murine plasma samples

Blood samples were collected from mice via exsanguination through the inferior vena cava (IVC) at the time of sacrifice and placed into tubes containing ethylenediaminetetraacetic acid (EDTA) to prevent coagulation. Following collection, the blood was centrifuged at 4,500g for 10 minutes, separating the plasma component, which was then stored at -80°C. SuPAR plasma levels were quantified using a manual enzyme-linked immunosorbent assay (ELISA) with a dilution factor of 1 to 4. The Mouse uPAR DuoSet ELISA kit from R&D Systems (Catalog #: DY531) was employed as per the manufacturer's instructions.

2.9 Single cell RNA sequencing of human liver

Liver tissue was collected during surgical intervention from two healthy donors and two patients with hepatitis B virus cirrhosis as described previously[5]. Informed consent was obtained from all the patients and donors. Liver tissue samples were quickly diluted in pre-cooled DMEM medium. Followed triple washing with PBS, tissues were cut into 1-3 mm^3 pieces, mixed with a cryopreservation solution (10% DMSO and 90% fetal bovine serum), and stored at -80°C. Aditionally single-cell transcriptomes of 5 healthy livers and 5 cirrhotic liver samples[6], and 3 cirrhotic liver samples[7] were obtained, that were described before. All datasets were processed using Seurats (v4.3.0) single-cell workflow. Cells with fewer than 200 or more than 2500 genes, or more than 15% mitochondrial content were excluded. Batch effects were minimized by using Seurat's integration anchors[8] based on 2000 highly variable

genes, and data were harmonized across batches via reciprocal principal component analysis.

2.10 Statistics

Statistical analysis was performed using SPSS® Version 29.0.0.0 (SPSS Inc., Chicago, IL). A two-sided p-value < 0.05 indicated statistical significance. For non-normally distributed values, mean and interquartile range (IQR) were calculated. Mann-Whitney U-test/Wilcoxon rank-sum test compared two independent groups, and Kruskal-Wallis test, with Bonferroni correction for multiple testing, compared multiple groups. While Spearman's rank correlation coefficient assessed the linear correlation of non-normally distributed metric or ordinal variables. Youden's J statistic was applied to the receiver operating characteristic (ROC) curve to determine the optimal threshold. Pearson chi-square test was used for testing of statistical differences between two categorical variables. To assess whether suPAR improves mortality and ACLF prediction beyond current risk stratification tools, multivariable logistic regression models were fitted with and without suPAR, and the resulting area under the curve (AUC) with their respective 95% confidence intervals (95% CI) were compared. For the derivation cohort a univariate logistic regression analysis was performed to evaluate individual variables, and those with significant associations were subsequently included in a multivariate logistic regression model. Backward elimination removed statistically insignificant variables. Additionally, a Cox proportional hazards model was used to calculate the hazard ratio over time in the validation cohort. Graphs were generated using PRISM® (GraphPad, USA) and BioRender APP (BioRender, Canada), tables were generated using Microsoft Word (Microsoft Corporation, USA).

Table S1: Primary antibodies used for Multiplex immune fluorescence staining

Target Antigen	Host	Dilution	Manufacturer	Catalog Nr.
uPAR	Rabbit	1/500	BIOSSUSA	BS-1927R
Hep Par-1	Mouse	1/500	Agilent	M715801-2
CD 45	Mouse	1/500	Agilent	M0701
CD 31	Rabbit	1/100	abcam	ab28364
IBA-1	Mouse	1/400	Sigma-Aldrich	MABN92
HNF4 alpha	Rabbit	1/1000	abcam	ab201460
CD 3	Mouse	1/200	DAKO	A0452
MPO	Rabbit	1/1000	abcam	ab208670
CK-19	Rat	1/200	DSHB biology	TROMA-III

Abbreviations: uPAR: urokinase Plasminogen Activator Receptor, Hep Par-1: Hepatocyte Specific Antigen, CD 45: Cluster of differentiation 45, Protein tyrosine phosphatase, leukocyte common antigen, CD 31: Cluster of differentiation 31, Platelet endothelial cell adhesion molecule-1, PECAM-1, IBA-1: ionized calcium-binding adapter molecule 1 (IBA1), Allograft inflammatory factor 1 (AIF-1), HNF4 alpha: Hepatocyte nuclear factor 4 alpha, CD 3: Cluster of differentiation 3, MPO: Myeloperoxidase, CK-19: Cytokeratin-19

Table S2: Secondary antibodies used for Multiplex immune fluorescence staining

Target Antigen	Fluorochrome	Host	Dilution	Manufacturer	Catalog Nr.
Mouse IgG (H+L) F(ab')2	Alexa Fluor® 488	Goat	1/500	Cell Signaling	4409
Mouse IgG (H+L) F(ab')2	Alexa Fluor® 647	Goat	1/500	Cell Signaling	4410
Rabbit IgG (H+L), F(ab')2	Alexa Fluor® 488	Goat	1/500	Cell Signaling	4412
Rabbit IgG (H+L), F(ab')2	Alexa Fluor® 647	Goat	1/500	Cell Signaling	4414
Rat IgG (H+L), F(ab')2	Alexa Fluor® 555	Goat	1/500	Cell Signaling	4417

Abbreviations: IgG: Immunoglobulin G, (H+L): heavy + light chain, F(ab')2: Antigen-binding fragment

Table S3: SuPAR levels as per organ failure in the derivation cohort

organ failure		definition	median suPAR levels	IQR suPAR levels	p-value				
					**1 - 2**	**1 - 3**	**2 - 3**		**overall**
	**liver failure**				0.002	<0.001	1.0		<0.001
	1	Bilirubin < 6 mg/dl	12.7 ng/ml	9.1 – 15.4 ng/ml					
	2	Bilirubin 6-12mg/dl	15.75 ng/ml	13.28 – 30.90 ng/ml					
	3	Bilirubin >12 mg/dl	20 ng/ml	13.90 – 31.70 ng/ml					
	**respiratory failure**								
	1	PaO2/FiO2 ratio > 300	14.00 ng/ml	10.80 – 18.33 ng/ml	0.04	0.004	0.799		0.004
	2	PaO2/FiO2 ratio 200 - 300	20.60 ng/ml	15.30 –					

				28.45 ng/ml					
	3	PaO2/FiO2 ratio ≤ 200 or mechanical ventilation	47.20 ng/ml	26.90 – 64.28 ng/ml					
	**coagulatory failure**				-	-	-		0.59
	1	INR < 2	13.60 ng/ml	10.60 – 17.20 ng/ml					
	2	INR 2 - < 2.5	20.15 ng/ml	13.45 – 24.58 ng/ml					
	3	INR ≥ 2.5	20.00 ng/ml	17.48 – 34.35 ng/ml					
	**renal failure**				0.266	0.003	0.374		0.003
	1	Creatinine < 2.0 mg/dl	14.10 ng/ml	10.80 – 18.30 ng/ml					

	2	Creatinine 2.0 - < 2.5 mg/dl	18.50 ng/ml	13.18 – 25.15 ng/ml					
	3	Creatinine ≥ 3.5 mg/dl or renal replacement therapy	38.90 ng/ml	23.40 – 64.98 ng/ml					
	**encephalopathic failure**				0.811	0.019	0.145		0.019
	1	West-Haven Grade for Hepatic Encephalopathy = 0	14.00 ng/ml	11.00 – 19.15 ng/ml					
	2	West-Haven Grade for Hepatic Encephalopathy = 1 – 2	20.30 ng/ml	14.75 – 31.35 ng/ml					
	3	West-Haven Grade for Hepatic Encephalopathy = 3 – 4	20.30 ng/ml	14.75 – 31.35 ng/ml					

	**circulatory failure**				0.014	0.008	0.639		0.008
	1	MAP ≥70 mmHg	14.55 ng/ml	11.75 – 20.60 ng/ml					
	2	MAP <70 mmHg	13.20 ng/ml	10.50 – 17.60 ng/ml					
	3	vasopressor therapy	17.90 ng/ml	11.70 – 36.40 ng/ml					

Kruskal–Wallis test with Bonferroni correction for multiple testing.

Abbreviations: suPAR: Soluble urokinase plasminogen activator receptor, IQR: Interquartile Range, PaO2, FiO2: inspiratory oxygen concentration, PaO2: arterial partial pressure of oxygen, INR: International normalized ratio, MAP: Mean Arterial Pressure

Table S4: ROC analysis for 90 days mortality in patients with decompensated cirrhosis

		AUC	CI 95%	p-value
AD score		0.800	0.660 - 0.940	0.000
MELD score		0.703	0.547 - 0.858	0.011
MELD-Na score		0.759	0.618 – 0.901	0.000
Child-Pugh Score (points)		0.721	0.599 – 0.842	0.000
SuPAR (ng/ml)		0.725	0.579 - 0.872	0.009
SuPAR ≥ 14.0 ng/ml		0.762	0.621 – 0.902	0.000
CRP (mg/dl)		0.516	0.331 – 0.702	0.864

AUROC analysis

Abbreviations: AUC: Area Under the Curve, AD score: CLIF-C Acute Decompensation score, MELD: Model for End-Stage Liver Disease, SuPAR: Soluble urokinase plasminogen activator receptor, CRP: C-reactive protein, 95% CI: 95% confidence interval, p: p-value

Table S5: ROC analysis for developing ACLF in patients with decompensated cirrhosis

		AUC	CI 95%	p-value
AD score		0.695	0.571 - 0.819	0.002
MELD score		0.740	0.617 - 0.863	0.000
MELD-Na score		0.727	0.602 – 0.852	0.000
Child-Pugh Score (points)		0.653	0.505 – 0.802	0.043
SuPAR (ng/ml)		0.673	0.528 - 0.812	0.014
SuPAR ≥ 14.7 ng/ml		0.621	0.485 – 0.758	0.081
CRP (mg/dl)		0.571	0.419 – 0.722	0.361

AUROC analysis

Abbreviations: AUC: Area Under the Curve, suPAR: Soluble urokinase plasminogen activator receptor, AD score: CLIF-C Acute Decompensation score, MELD: Model for End-Stage Liver Disease, CRP: C-reactive protein, 95% CI: 95% confidence interval, p: p-value

Table S6: Logistic regression models using a suPAR cut-off of 14.0 ng/ml for prediction of 90-day mortality in patients with decompensated liver cirrhosis in the derivation cohort

Score				Score + suPAR		
**Variables included**	**AUC**	**CI 95%**		**Variables included**	**AUC**	**CI 95%**
MELD-Na score	0.759	0.618 – 0.901		Sodium + Bilirubin + Creatinine + INR + suPAR	0.812	0.679 – 0.945
Child-Pugh Score (points)	0.728	0.572 – 0.883		Child-Pugh-Score (points) + suPAR	0.790	0.646 – 0.935
MELD	0.703	0.547 – 0.858		MELD + suPAR	0.785	0.639 – 0.931
AD Score	0.800	0.660 – 0.940		AD score + suPAR	0.843	0.728 – 0.958

AUROC analysis

Abbreviations: AUC: Area Under the Curve, MELD: Model for End-Stage Liver Disease, SuPAR: Soluble urokinase plasminogen activator receptor, 95% CI: 95% confidence interval, INR: International normalized ratio

Table S7: Logistic regression models using a suPAR cut-off of 14.7 ng/ml for prediction of ACLF in patients with decompensated liver cirrhosis in the derivation cohort

**Score**				**Score + suPAR**		
**Variables included**	**AUC**	**CI 95%**		**Variables included**	**AUC**	**CI 95%**
MELD-Na score	0.727	0.602 – 0.852		Sodium + Bilirubin + Creatinine+ INR + suPAR	0.755	0.635 – 0.876
Child-Pugh Score (points)	0.653	0.505 – 0.802		Child-Pugh-Score (points) + suPAR	0.670	0.509 – 0.830
MELD	0.740	0.617 - 0.863		MELD + suPAR	0.741	0.615 – 0.867
AD Score	0.695	0.571 - 0.819		AD score + suPAR	0.744	0.626 – 0.862

AUROC analysis

Abbreviations: AUC: Area Under the Curve, MELD: Model for End-Stage Liver Disease, SuPAR: Soluble urokinase plasminogen activator receptor, 95% CI: 95% confidence interval, INR: International normalized ratio

Table S8: Patients in the derivation cohort analysed according to patient sex

		Female (n=65)	Male (n=107)	p-value (male-female)
**Number of patients (%)**		37.8%	62.2%	
**Age (years) median (IQR)**		49 (41-59.5)	53 (46-61)	0.201
**Etiology**				0.519
	ALD (%)	32 (53.3%)	59 (57.3%)	
	viral (%)	4 (6.7%)	15 (14.6%)	
	MASLD (%)	5 (8.3%)	6 (5.8%)	
	cryptogenic (%)	1 (1.7%)	4 (3.9%)	
	multiple (%)	9 (15.0%)	13 (12.6%)	
	other/ rare (%)	9 (15.0%)	6 (5.8%)	
	no data	5 (7.7%)	4 (3.7%)	
**Laboratory and clinical parameters**				
	SuPAR (ng/ml) median (IQR)	13.6 (9.3-17.8)	14.3 (10.6-20.6)	0.314
	WBC (10^9/l) median (IQR)	8 (4.8-11.1)	8.65 (5.7-12.4)	0.365
	Platelets (10^9/l) median (IQR)	144 (85-209)	89 (60-159)	0.006
	ALT (U/l) median (IQR)	37 (25-63.5)	37 (24-73)	0.633
	ALP (U/l) median (IQR)	162 (127-220)	141 (91-210)	0.074
	Albumin (g/l) median (IQR)	30 (26-34)	30 (25-34)	0.532
	CRP (mg/dl) median (IQR)	15 (5-43)	24 (7-64)	0.162

	Hemoglobin (g/l) median (IQR)	95 (79-115)	98 (87-113)	0.334
	Sodium (mmol/l) median (IQR)	136 (132-139)	136 (131-140)	0.943
	Potassium (mmol/l) median (IQR)	3.9 (3.35-4.4)	3.8 (3.6-4.4)	0.830
	Creatinine (µmol/l) median (IQR)	69 (49.25-109.5)	76 (60-108)	0.227
	Bilirubin (µmol/l) median (IQR)	87 (25-191)	108 (39-207)	0.227
	INR median (IQR)	1.6 (1.4-1.9)	1.6 (1.4-2.0)	0.948
	MAP (mmHg) median (IQR)	79 (70-83.83)	86.67 (77-95.83)	0.019
**Disease dynamics**				
	hospitalization (days) median (IQR)	14 (8-24)	11 (6-22)	0.370
	ICU treatment	25.5%	21.6%	0.595
	90-days-mortality (%)	20%	15%	0.393

Pearson chi-square test for categorical data or the Mann–Whitney U/Wilcoxon rank-sum test for numerical data.

Abbreviations: suPAR: soluble Urokinase Plasminogen Activator Receptor, IQR: interquartile range, ALD: alcoholic liver disease, MASLD: metabolic dysfunction associated liver disease, ICU: intensive care unit, WBC: white blood cell count, ALT: alanine aminotransferase, ALP: alkaline phosphatase, CRP: C-reactive protein, ICU:

intensive care unit, INR: International normalized ratio, MAP: Mean arterial pressure, n. a.: not applicable

Table S9: Patients in the derivation cohort analyzed according to etiology of liver cirrhosis

	ALD (n=91)	viral (n=19)	MASLD (n=11)	cryptogenic (n=5)	multiple (n=22)	other / rare (n=15)	no data (n=9)	p-value (ALD-rest)
**Number of patients (%)**	55.8 %	11.7 %	6.7%	3.1%	13.5%	9.2%	5.2%	
**Age (years) median (IQR)**	51 (45-60)	53 (45-57)	67 (49-72)	61 (49-72)	47 (37-55)	50 (36-57)	55 (42-64)	0.985
**Laboratory and clinical parameters**								
SuPAR (ng/ml) median (IQR)	14.6 (10.9-22.7)	12.1 (6.7-20.7)	11.6 (8.1-13.7)	15.8 (8.1-16.9)	14.5 (12.2-18.1)	14.0 (12.4-18.6)	9.9 (6.0-18.5)	0.086
WBC (10^9/l) median (IQR)	8.8 (5.8-13.5)	6.9 (5.2-9.5)	7.8 (5.4-10.3)	3.8 (0.9-5.0)	7.6 (4.9-10.9)	8.9 (6.4-17.1)	9.0 (4.7-16.6)	0.100

	Platelets (10^9/l) median (IQR)	116 (75-182)	124 (65-281)	144 (61-202)	45 (13-182)	65 (42-119)	119 (100-258)	99 (60-172)	0.267
	ALT (U/l) median (IQR)	34 (23-65)	43 (31-147)	24 (12-36)	14 (11-34)	43 (27-67)	81 (49-173)	22 (14-59)	0.165
	ALP (U/l) median (IQR)	160 (115-217)	130 (80-170)	98 (75-209)	128 (47-342)	138 (108-178)	220 (130-360)	114 (67-154)	0.392
	Albumin (g/l) median (IQR)	30 (25-34)	32 (27-40)	34 (30-41)	34 (24-36)	29 (23-34)	30 (27-34)	30 (25-31)	0.420
	CRP (mg/dl) median (IQR)	25 (8-63)	26 (2-44)	17 (8-31)	43 (10-71)	9 (5-45)	15 (0-28)	13 (0-35)	0.078
	Hemoglobin (g/l) median (IQR)	95 (83-113)	107 (89-113)	115 (102-118)	79 (78-105)	98 (78-106)	110 (100-122)	95 (81-114)	0.065
	Sodium (mmol/l)	136 (131-139)	138 (132-142)	131 (128-136)	140 (136-144)	136 (130-141)	136 (130-138)	136 (130-139)	0.791

	median (IQR)								
	Potassium (mmol/l) median (IQR)	3.7 (3.4-4.4)	4.2 (3.5-4.8)	3.9 (3.5-4.7)	3.8 (3.7-4.9)	4.1 (3.6-4.4)	3.9 (3.6-4.3)	3.5 (3.2-4.1)	0.176
	Creatinine (µmol/l) median (IQR)	71 (57-100)	77 (52-159)	91 (74-105)	170 (65-102)	84 (49-121)	63 (49-92)	63 (40-168)	0.630
	Bilirubin (µmol/l) median (IQR)	102 (34-221)	128 (12-413)	33 (17-83)	39 (28-76)	84 (35-136)	168 (79-277)	73 (18-356)	0.581
	INR median (IQR)	1.7 (1.4-2.0)	1.8 (1.4-2.1)	1.5 (1.1-2.0)	1.4 (1.4-1.8)	1.8 (1.4-2.1)	1.6 (1.4-1.7)	1.6 (1.4-1.8)	0.271
	MAP (mmHg) median (IQR)	84.33 (75.66-94.66)	90 (84.66-104)	73.33 (71.33-80.66)	66.66 (62.66-78.17)	85.67 (75.17-97)	77.67 (66.67-96.67)	88.17 (83.08-99.8)	0.573
**Disease dynamics**									

	hospitalization (days) median (IQR)	10 (7-22)	14 (7-21)	15 (7-21)	16 (11-18)	17 (6-30)	14 (6-36)	12 (7-17)	0.245
	ICU treatment	25.6 %	27.3 %	20.0%	0.0%	22.2%	6.7%	42.9 %	0.220
	90-days-mortality (%)	16.5 %	10.5 %	9.1%	0.0%	22.7%	26.7 %	22.2 %	0.975

Pearson chi-square test for categorical data or the Mann–Whitney U/Wilcoxon rank-sum test for numerical data.

Abbreviations: suPAR: soluble Urokinase Plasminogen Activator Receptor, IQR: interquartile range, ALD: alcoholic liver disease, MASLD: metabolic dysfunction associated liver disease, ICU: intensive care unit, WBC: white blood cell count, ALT: alanine aminotransferase, ALP: alkaline phosphatase, CRP: C-reactive protein, ICU: intensive care unit, INR: International normalized ratio, MAP: Mean arterial pressure, n. a.: not applicable

Fig. S1: KEGG analysis for function of PLAUR in uPAR expressing cells in HBV cirrhosis

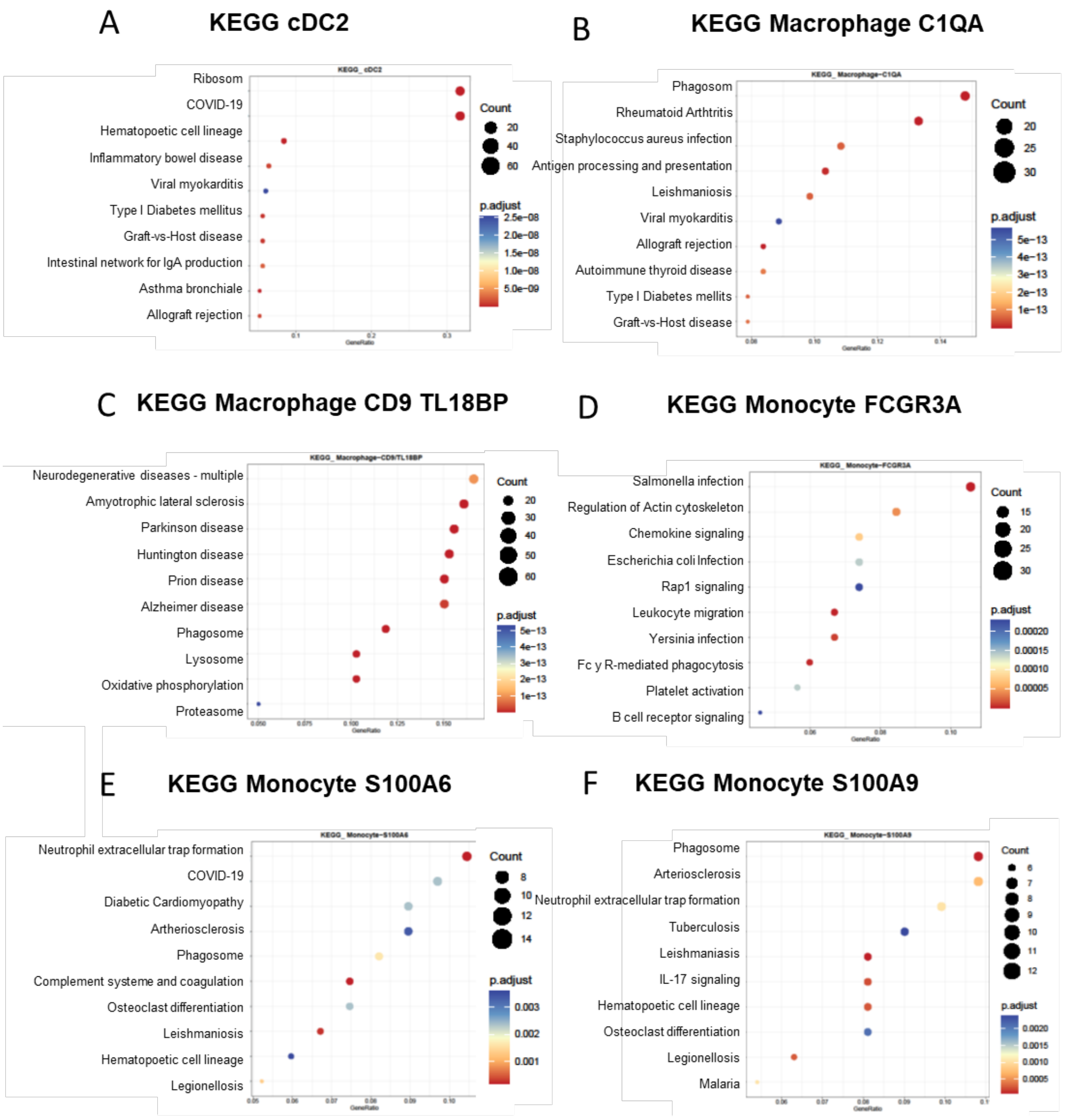

Abbreviations: KEGG: Kyoto Encyclopedia of Genes and Genomes analysis, PLAUR - Plasminogen Activator Urokinase Receptor, cDC2: Conventional Dendritic Cells, subtype 2, C1QA: complement component 1q subcomponent A, CD9: Cluster of Differentiation 9, TL18: Tetraspanin-18, TL18BP: Tetraspanin-18 binding protein, FCGR3A: Fc gamma receptor III-A (CD16a), S100A6: S100 calcium-binding protein A6, S100A9: S100 calcium-binding protein A9

Supplementary references

1. Moreau R, Jalan R, Gines P, et al. Acute-on-chronic liver failure is a distinct syndrome that develops in patients with acute decompensation of cirrhosis. *Gastroenterology*. 2013;144(7):1426-1437, 1437.e1-9. doi:10.1053/j.gastro.2013.02.042

2. WMA - The World Medical Association-WMA Declaration of Helsinki – Ethical Principles for Medical Research Involving Human Subjects. Accessed August 8, 2023. https://www.wma.net/policies-post/wma-declaration-of-helsinki-ethical-principles-for-medical-research-involving-human-subjects/

3. Percie du Sert N, Hurst V, Ahluwalia A, et al. The ARRIVE guidelines 2.0: Updated guidelines for reporting animal research. *PLoS Biol*. 2020;18(7):e3000410. doi:10.1371/journal.pbio.3000410

4. Guillot A, Kohlhepp MS, Bruneau A, Heymann F, Tacke F. Deciphering the Immune Microenvironment on A Single Archival Formalin-Fixed Paraffin-Embedded Tissue Section by An Immediately Implementable Multiplex Fluorescence Immunostaining Protocol. *Cancers*. 2020;12(9):2449. doi:10.3390/cancers12092449

5. Bai Q, Hong X, Lin H, et al. Single-cell landscape of immune cells in human livers affected by HBV-related cirrhosis. *JHEP Rep Innov Hepatol*. 2023;5(11):100883. doi:10.1016/j.jhepr.2023.100883

6. Ramachandran P, Dobie R, Wilson-Kanamori JR, et al. Resolving the fibrotic niche of human liver cirrhosis at single-cell level. *Nature*. 2019;575(7783):512-518. doi:10.1038/s41586-019-1631-3

7. Zhang Q, He Y, Luo N, et al. Landscape and Dynamics of Single Immune Cells in Hepatocellular Carcinoma. *Cell*. 2019;179(4):829-845.e20. doi:10.1016/j.cell.2019.10.003

8. Stuart T, Butler A, Hoffman P, et al. Comprehensive Integration of Single-Cell Data. *Cell*. 2019;177(7):1888-1902.e21. doi:10.1016/j.cell.2019.05.031
